# Foxn1 expression in keratinocytes is stimulated by hypoxia: further evidence of its role in skin wound healing

**DOI:** 10.1038/s41598-018-23794-5

**Published:** 2018-04-03

**Authors:** Anna Kur-Piotrowska, Joanna Bukowska, Marta M. Kopcewicz, Mariola Dietrich, Joanna Nynca, Mariola Slowinska, Barbara Gawronska-Kozak

**Affiliations:** Institute of Animal Reproduction and Food Research, Polish Academy of Sciences, Olsztyn, Poland

## Abstract

Recent studies have shown that the transcription factor Foxn1, which is expressed in keratinocytes, is involved in the skin wound healing process, yet how Foxn1 functions remains largely unknown. Our latest data indicate that Foxn1 drives skin healing via engagement in re-epithelization and the epithelial-mesenchymal transition (EMT) process. In the present study, 2D-DIGE proteomic profiling analysis of *in vitro* cultured keratinocytes transfected with adenoviral vector carrying Foxn1-GFP or GFP alone (control) revealed forty proteins with differential abundance between the compared groups. Among the proteins with Foxn1-dependent expression, several enable adaptation to hypoxia. Subsequent experiments revealed that hypoxic conditions (1% O_2_) stimulate endogenous and exogenous (transfected Ad-Foxn1) Foxn1 expression in cultured keratinocytes. A proteomics analysis also identified proteins that can act as a factors controlling the balance between cell proliferation, differentiation and apoptosis in response to Foxn1. We also showed that in C57BL/6 keratinocytes, the stimulation of Foxn1 by hypoxia is accompanied by increases in Mmp-9 expression. These data corroborate the detected co-localization of Foxn1 and Mmp-9 expression *in vivo* in post-wounding skin samples of Foxn1::Egfp transgenic mice. Together, our data indicate that Foxn1 orchestrates cellular changes in keratinocytes in both physiological (self-renewal) and pathological (skin wound healing) contexts.

## Introduction

Skin is a multi-layered organ composed of an epidermis (the external stratified epithelium), the underlying dermis, and the hypodermis, which is built up by subcutaneous adipose tissue^1,2^. As the largest organ of the body, skin fulfils multiple key functions, acting as a protective barrier against environmental insults, an immunological response system and a neuro-endocrine organ; these functions all require complex skin reactivity^1,2^.

Skin wound healing is a highly coordinated, multi-phase process that involves inflammation, re-epithelialization and remodelling resulting in scar formation^3^. Keratinocyte migration is essential for re-epithelialization and for proper wound healing. A number of factors and molecular pathways affect keratinocyte mobilization, although most of them remain poorly understood^4,5^. Increasing evidence suggests that oxygen plays a critical role in re-epithelialization and wound healing, directing the outcome of the process to scar formation, scarless healing (regeneration) or failure to heal, although the involvement of hypoxia response factors and their downstream effectors is not fully recognized^6,7^.

We have recently shown that the forkhead box N1 (Foxn1) transcription factor is a key regulator of skin wound healing; it is involved in the re-epithelialization and remodelling phases, most likely due to its role in promoting epithelial-to-mesenchymal transition (EMT)^8,9^. Moreover, our recent data imply that Foxn1 is a pivotal control element of skin development and maturation^10^.

In mice, an inactivating mutation in Foxn1 has a pleiotropic effect, resulting in a nude phenotype^11^. One unique feature of adult nude mice is their capacity for skin regeneration^12,13^. Similar to the mammalian foetus, an established model of skin regeneration, nude mice display characteristics associated with scarless healing after skin wounding^12–14^. Interestingly, both mammalian foetuses and nude mice lack Foxn1 activity in the skin, which is required to complete the regenerative wound healing process. A recently performed comparison of uninjured mouse foetus skin collected during the regenerative period (14th day of embryonic development; Foxn1 inactive) and nude mice (Foxn1-deficient) revealed similarities in transcriptomic signatures that predispose both models to regenerative skin healing when wounding occurs^10^. We also proposed that Foxn1 expression in the skin is an essential condition for establishing the adult skin phenotype and that a lack of Foxn1 maintains skin in a state of neoteny (i.e., an immature stage of development)^10^.

Furthermore, this analysis revealed that although total skin tissues from nude mice displayed the up-regulation of matrix metalloproteinase 9 (Mmp-9) in comparison to control mice, enzymatically separated epidermis (Foxn1 host tissues) from nude skin showed significant down-regulation of Mmp-9 expression. In the skin, Mmp-9 promotes the migration of epithelial cells^15^, among other functions, and its expression is regulated by a number of factors. However, the complexity of Mmp-9 action in various physiological and pathological conditions suggests that there are as-yet undiscovered pathways involved in its regulation and function^16^.

The differences in skin gene expression profile detected between nude and wild-type mice, as well as the involvement of Foxn1 in skin wound healing, led us to the present study. In this manuscript, we analysed whether transient activation of Foxn1 in primary cultures of mouse keratinocytes (i) stimulates Mmp-9 expression, (ii) controls the differentiation and migration ability of keratinocytes, and (iii) regulates the protein expression profile of keratinocytes, as well as whether hypoxic conditions affect Foxn1 expression.

We revealed for the first time that Foxn1 expression in primary cultures of keratinocytes is induced by hypoxia. This induction was detected for endogenous Foxn1 (in Foxn1-positive keratinocytes from C57BL/6 (B6) mice) and for Foxn1 that was exogenously introduced into nude (Foxn1-deficient) keratinocytes. We also showed that Foxn1 stimulates keratinocyte differentiation. Moreover, we report that Foxn1 activation increased the levels of pro-apoptosis and hypoxia-regulated proteins. In addition, our data confirmed that Mmp-9 expression in keratinocytes is stimulated by hypoxia.

## Results and Discussion

Recently, we demonstrated that Foxn1 is involved in skin wound healing through its participation in re-epithelialization and EMT^8^. Using a Foxn1::Egfp (enhanced green fluorescent protein) transgenic mouse model, we showed that Foxn1-eGFP-positive keratinocytes accumulate at wound margins, form a leading epithelial tongue that migrates under the scab, and finally shape the entire length of the neo-epidermis covering the wounded area. In sum, in the present study, we reasoned that Foxn1 controls reparative skin wound healing through the induction of Mmp-9 expression in keratinocytes and regulates keratinocyte differentiation and/or migration ability.

### Foxn1 and Mmp-9 expression in injured skin and keratinocytes

**Figure 1:**
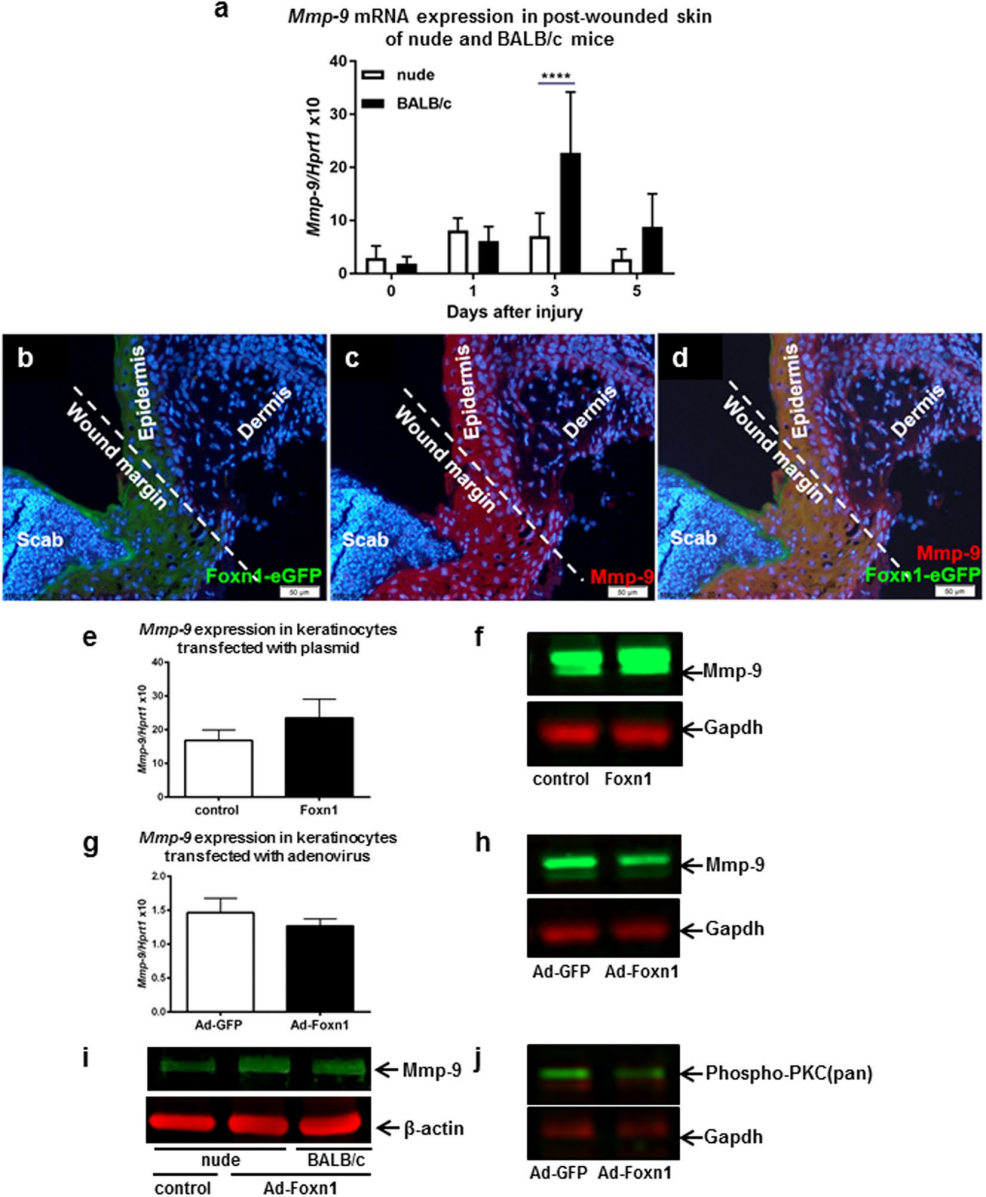
Mmp-9 is expressed in mice injured skin tissues (**a–d**) and in keratinocytes (**e–j**). *Mmp-9* mRNA expression in the skin of nude (Hsd:Athymic Nude-Foxn1^nu^) and BALB/c mice at post-wounding days 1, 3, and 5 (**a**). Immunofluorescent detection of Foxn1-eGFP (**b**) and Mmp-9 (**c**) and co-localization of Foxn1-eGFP and Mmp-9 signals (**d**) in the skin of Foxn1::Egfp mice at day 2 after injury. *Mmp-9* mRNA (**e**,**g**) and corresponding Mmp-9 protein (**f**,**h**) expression in keratinocytes isolated from B6 (E-H, J), BALB/c (**i**) and nude (**i**) mice and co-cultured with dermal fibroblasts (DFs). Keratinocytes were transfected with Foxn1-containing plasmid (**e**,**f**) or adenovirus (Ad-Foxn1; **g–j**) and cultured for 48 h. Control cultures were transfected with a vector expressing GFP alone. Representative Western blot analysis of phospho-PKC (pan) protein expression in Ad-Foxn1- or Ad-GFP-transfected keratinocytes co-cultured with DFs (**j**). Full-length blots and densitometric analysis are presented in Supplementary Fig. S1. (**b–d**) Scale bar 50 µm. Values are the mean ± SD, ****p < 0.0001. (**a**) Modified from: Scarless skin wound healing in FOXN1-deficient (nude) mice is associated with distinctive matrix metalloproteinase expression. Gawronska-Kozak B., Matrix Biol 2011;30(4):290–300^12^, with permission of Elsevier.

Mmp-9 is weakly expressed in unwounded skin tissues, but its expression is greatly elevated during the first days after injury in control (wild-type) mice^12,17^. In contrast, injured skin of Foxn1-deficient (nude) mice respond to trauma with a minor up-regulation of *Mmp-9* mRNA expression, as observed at days 3–5 after wounding (Fig. 1a)^12^. To explore Foxn1 and Mmp-9 localization, skin tissues from Foxn1::Egfp mice were collected at post-wounding day 2^8^. A robust endogenous eGFP signal indicating Foxn1 expression was detected in the thickened epidermis bordering the injury site, thick epidermis at the wound site and compacted cells migrating underneath the scab (Fig. 1b). The same sections were stained for Mmp-9 expression (Fig. 1c). Mmp-9 protein localized to the epidermis adjacent to the wound site, to the newly forming epithelial tongue underneath the scab, and to some cells in the dermal part of the skin (Fig. 1c). The robust Foxn1-eGFP signal at the epidermal part of the skin overlaps with Mmp-9 expression, revealing the co-localization of Foxn1 and Mmp-9 in the epidermis at the injury site (Fig. 1d).

Next, we aimed to examine whether Foxn1 is involved in the regulation of Mmp-9 expression in keratinocytes. To this end, we used plasmid or adenoviral overexpression systems. Primary cultures of keratinocytes isolated from B6 mice were transfected with vectors carrying cDNA encoding Foxn1-GFP or GFP alone (control). Considering that the interaction between keratinocytes and fibroblasts strongly influences the expression of Mmp-9^18^, we performed a series of independent experiments using two different vectors, plasmid (Fig. 1e,f) and adenovirus (Fig. 1g,h,i,j), in a keratinocyte-dermal fibroblast (DFs) co-culture system. The experiments yielded ambiguous results. The replicate experiments showed either an increase in *Mmp-9* mRNA expression or a lack of significant difference in *Mmp-9* mRNA expression between keratinocytes transfected with Foxn1 and those transfected with GFP (control), regardless of the vector used (plasmid, Fig. 1e; adenovirus, Fig. 1g). Western blot assays for Mmp-9 protein expression also produced equivocal results. The forced overexpression of Foxn1 in infected keratinocytes either induced Mmp-9 expression (Fig. 1f, and see Supplementary Figure S1A) or produced only slight differences in comparison with Ad-GFP (control)-treated keratinocytes (Fig. 1h, and see Supplementary Figure S1A). Next, we transfected keratinocytes isolated from nude (Foxn1-deficient) mice and their counterpart BALB/c (Foxn1-active) mice with Ad-Foxn1 or Ad-GFP (Fig. 1i, and see Supplementary Figures S1B and S1C). We detected a slight but not statistically significant increase in Mmp-9 protein expression in Ad-Foxn1-transfected keratinocytes from both the nude and BALB/c backgrounds (Fig. 1i, and see Supplementary Figures S1B and S1C). To ensure that the Foxn1 introduced into keratinocytes had sustained activity, we analysed the expression of phospho-PKC (pan), which is recognized as a downstream target of Foxn1 in keratinocytes. Western blot analysis demonstrated that Foxn1 overexpression suppressed the levels of PKC family proteins (Fig. 1j, and see Supplementary Figure S1D), as previously shown^19^.

### Foxn1 regulates keratinocyte differentiation and migration

Healthy epidermis undergoes continual renewal, which involves the proliferation and differentiation of keratinocytes. The expression of specific keratins can be used to define the status of keratinocytes in the skin. Keratinocytes in the basal layer, which are mitotically active and characterized by continual cell renewal, express keratin 14 (K14). Keratin 10 (K10) expression is common for cells in the suprabasal layer, indicating a withdrawal from the cell cycle and the initiation of differentiation^20^. Terminal differentiation in the spinous layer of the epidermis is marked by an increase in involucrin expression. Pathological states, e.g., in skin injury, trigger the process of keratinocyte activation, which is characterized by hyperproliferation and migration to cover the wounded area. The expression of specific keratins, namely, keratin 6, 16 and 17, has been used as a marker for activated keratinocytes^21^.

**Figure 2:**
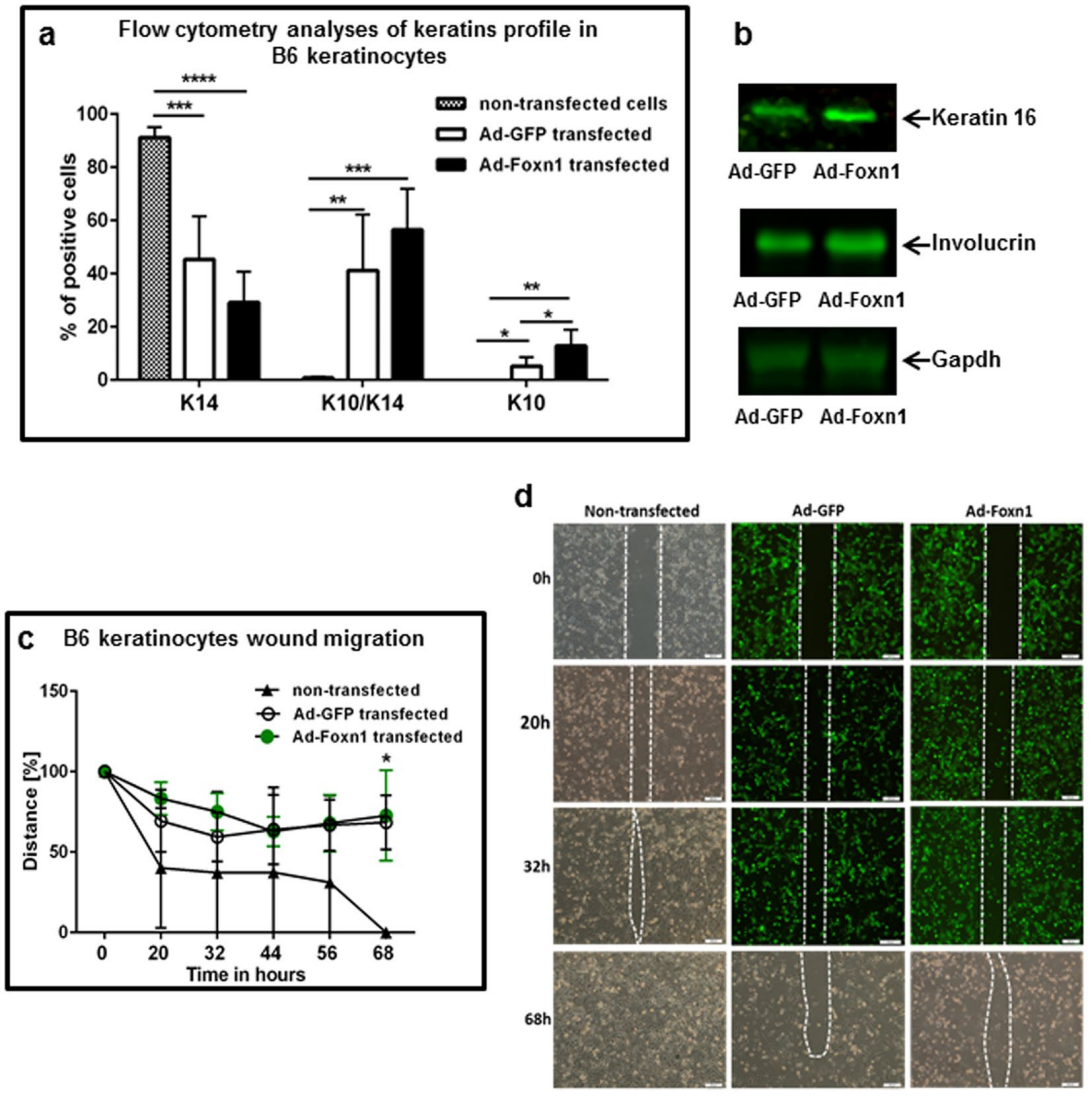
Foxn1 regulates keratinocyte differentiation and migration. Flow cytometric analysis of keratinocytes: non-transfected and transfected with Ad-Foxn1 or Ad-GFP, showing the percentage expressing keratin 14 (K14) only, keratin 10/keratin 14 (K10/K14) and K10 only (**a**). Representative Western blots of keratin 16 and involucrin protein expression in keratinocytes transfected with Ad-Foxn1 or Ad-GFP (control) (**b**). Full-length blots are presented in Supplementary Figure S2. Migratory abilities after pre-treatment with mitomycin C and wounding in monolayer cultures were analysed in non-transfected B6 keratinocytes and in keratinocytes transfected with Ad-Foxn1 or Ad-GFP (**c**,**d**). Representative images were taken at 0, 20, 32 and 68 h (**d**). Dotted lines indicate the distance between migrating keratinocytes (**d**). Migration is expressed as the percentage of the distance between the unclosed edges (**c**). Each well was prepared in duplicate, and the experiment was repeated two times (n = 11 animals). Values are the mean ± SD. Asterisks indicate significant differences (*p < 0.05; **p < 0.01; ***p < 0.001; ****p < 0.0001).

Flow cytometric analysis of cultured keratinocytes that were non-transfected or transfected with Ad-Foxn1 or Ad-GFP (control) were analysed for the expression of K14 and K10 markers of basal (mitotically active) and suprabasal (differentiated) layers, respectively (Fig. 2a). A significant increase in the percentage of K10-positive cells was detected in Ad-Foxn1-transfected cells (Fig. 2a; p < 0.05 vs Ad-GFP, p < 0.01 vs non-transfected). Keratinocytes that were double-positive for K14 and K10 reached a level of 56.46 ± 15.41% of the population for Ad-Foxn1, 41.16 ± 21.13% for Ad-GFP-transfected and 0.78 ± 0.56% for non-transfected cells. The largest proportion of K14-positive keratinocytes was observed in non-transfected cells (91.23 ± 3.83%), followed by Ad-GFP- (45.38 ± 16.17%) and Ad-Foxn1 (29.08 ± 11.64%)-transfected cells. Nevertheless, the increase in the percentage of K10/K14 and K10 among Ad-Foxn1-transfected cells indicates the stimulatory role of Foxn1 in the keratinocyte differentiation process. On the other hand, the data suggest that the process of transfection per se, regardless of the element transfected (i.e., Ad-Foxn1 or Ad-GFP), triggers changes in the percentage of the cells positive for K14 and K10 (Fig. 2a). Additionally, involucrin, the late marker of keratinocyte differentiation, and keratin 16, the marker of the keratinocyte activation stage, were up-regulated in Ad-Foxn1-transfected cells (Fig. 2b, and see Supplementary Figures S2A and B). Altogether, the protein profile of Foxn1-transfected cells indicates an essential role for Foxn1 in the acquisition of the terminal, mature keratinocyte phenotype.

On the functional basis (*in vitro* wound healing assay), our data revealed no differences in migratory ability between Ad-Foxn1- and Ad-GFP-transfected cells (Fig. 2c and d). However, non-transfected keratinocytes displayed higher motility compared to the transfected cells (p < 0.05, Fig. 2c and d). Our previous *in vivo* data showed that Foxn1-positive keratinocytes are engaged in the process of re-epithelialization, and Foxn1 co-localizes with Mmp-9 (see Fig. 1b–d) and keratin 16 in the wound margin and leading epithelial tongue^8,9^. Injury response *in vivo* is orchestrated by signals originating from wound-affected cells: keratinocytes, dermal fibroblasts, endothelial cells, and melanocytes. Our *in vitro* model included only keratinocytes and did not provide all the components essential for complex injury response, which may be one factor in the contrast observed between *in vitro* and *in vivo* data.

### Proteomic analysis of keratinocytes overexpressing the transcription factor Foxn1

The ambiguous results related to Mmp-9 expression spurred us to investigate the changes in global protein expression in keratinocytes that resulted from the activation of Foxn1 after Ad-Foxn1 transfection. We compared the proteomes of Foxn1-transfected and control keratinocytes using a two-dimensional difference gel electrophoresis (2D-DIGE) approach (see Supplementary Figure S3). Differentially labelled Ad-Foxn1-treated and control (Ad-GFP) samples were mixed and resolved within the same gel, and we were able to identify sixty-six of sixty-nine distinct protein spots (see Supplementary Figure S3 and Table S1). The list of spots was simplified to forty different proteins (Table 1), as some of the identified polypeptides occurred in several isoforms; for example, five different isoforms of 3-phosphoinositide-dependent protein kinase 1 (Pdpk1) were identified (see Supplementary Table S1). Almost all of the detected proteins were components of a network of predicted functional associations, as indicated by an analysis using the STRING database (see Supplementary Figure S4). Functional profiling of proteins with differential abundance in the proteomes of keratinocytes transfected with Ad-Foxn1 and Ad-GFP was performed with g:Profiler^22^. The characterization indicated that some of these proteins are involved in the cellular response to stress, carboxylic acid metabolic processes or biological adhesion (see Supplementary Table S3). Interestingly, all of these functions may be involved in the wound healing context.

**Table 1:** Simplified list of proteins with differential abundance in keratinocytes transfected with Ad-Foxn1 and control keratinocytes (transfected with Ad-GFP).

No.	Protein name	Symbol	The highest av. ratio^*^
Up-regulated in Ad-Foxn1 transfected keratinocytes			
1	mitochondrial aconitate	Aco2	1.24
2	enoyl-CoA hydratase, mitochondrial precursor	Echs1	1.23
3	1-phosphatidylinositol 4,5-bisphosphate phosphodiesterase delta-1 isoform X1	Plcd1	1.33
4	heat shock 70 kDa protein 1B	Hspa1a	1.86
5	type II keratin 5	Krt5	1.41
6	keratin, type II cytoskeletal 6 A	Krt6a	1.35
7	Krt6b protein	Krt6b	1.35
8	prolyl 4-hydroxylase, beta polypeptide, isoform CRA_a	P4hb	1.09
9	cathepsin D precursor	Ctsd	1.34
10	26 S proteasome non-ATPase regulatory subunit 13	Psmd13	1.22
11	malate dehydrogenase, cytoplasmic isoform Mdh1	Mdh1	1.11
12	annexin A8 isoform 1	Anxa8	2.06
13	PREDICTED: voltage-dependent anion-selective channel protein 1	Vdac1	1.19
14	thioredoxin	Txn	3.55
15	galectin-7	Lgals7	1.81
16	PREDICTED: elongation factor 1-delta isoform X9	Eef1d	1.19
Up-regulated in Ad-GFP transfected (control) keratinocytes			
1	PREDICTED: major vault protein isoform X1	Mvp	1.18
2	PREDICTED: alpha-actinin-4 isoform X4 [Mus musculus]	Actn4	1.20
3	gelsolin isoform 2	Gsn	1.21
4	caldesmon	Cald1	1.48
5	Mosin protein, partial	Msn	1.35
6	3-phosphoinositide-dependent protein kinase 1 isoform 1	Pdpk1	8.21
7	heat shock protein HSP 90-alpha	Hsp90aa1	1.18
8	heat shock protein HSP27	Hspb1	1.13
9	plastin 3 (T-isoform), isoform CRA_b, partial	Pls3	1.45
10	keratin, type II cytoskeletal 7	Krt7	1.37
11	D-3-phosphoglycerate dehydrogenase	Phgdh	1.22
12	perilipin-3	Plin3	1.14
13	6-phosphogluconate dehydrogenase, decarboxylating	Pgd	1.23
14	plasminogen activator inhibitor 2, macrophage	Serpinb2	1.46
15	PDZ and LIM domain protein 1	Pdlim1	1.12
16	arginase-1	Arg1	1.85
17	PREDICTED: poly(rC)-binding protein 1	Pcbp1	1.26
18	PREDICTED: LIM and SH3 domain protein 1 isoform X2	Lasp1	1.29
19	annexin A1	Anxa1	1.48
20	annexin III	Anxa3	1.62
21	annexin A4	Anxa4	1.36
22	prohibitin	Phb	1.90
23	phosphoglycerate mutase 1	Pgam1	1.18
24	serpin B8 isoform 1	Serpinb8	1.25

*For a full list of results obtained for particular isoforms, see Table S1.

Proteomics analysis did not confirm differences in Mmp-9 or PKC expression, as revealed by Western blot (compare Fig. 1f,h,i and j). The discrepancy may result from the different sensitivities of Western blot and 2D-DIGE. The application of specific antibodies and fluorescent detection seems to be more accurate than 2D-DIGE for the detection of Mmp-9 and PKC. The detection limit of 2D-DIGE might be lower because it is possible for Mmp-9 and PKC to be present in several proteoforms, which affects signal intensity. Moreover, Mmp-9 and PKC may be masked by the presence of abundant high molecular weight proteins with similar physicochemical characteristics (molecular weight and pI) in keratinocytes, which prevent their detection in 2D-DIGE.

Next, we compared the proteomics results with the differential transcriptome profiling of the epidermis and skin, contrasting Foxn1-deficient nude mice (model of regenerative skin wound healing) to B6 mice with active Foxn1 (model of reparative skin wound resolution), obtained in our previous study^10^. We are aware that the proteomics results (*in vitro* experiments) cannot be simply compared to the *in vivo* data from skin/epidermis transcriptome profiling. Nevertheless, the comparison revealed 14 molecules common to both analyses (Table 2), which is a substantial number considering that the proteomic data showed only forty differentially regulated proteins with altered abundances between the compared groups. These data confirmed the reliability of our results and, more importantly, highlighted the importance of Foxn1 in the regulation of those 14 proteins. Moreover, in agreement with the literature^23,24^, our results showed that 1-phosphatidylinositol 4,5-bisphosphate phosphodiesterase delta-1 (Plcd1) and galectin-7 (Lgals7) are up-regulated by Foxn1 (Table 1, and see Supplementary Table S1).

**Table 2:** Proteins identified both in proteomic analysis as differentially abundant in keratinocytes transfected with Ad-Foxn1 and keratinocytes transfected with Ad-GFP and among genes selected from sequencing data (skin samples of Foxn1-deficient nude mice vs Foxn1-active control mice).

Proteins identified both in proteomic analysis as differentially abundant in keratinocytes transfected with Ad-Foxn1 and keratinocytes transfected with Ad-GFP and among genes selected from sequencing data (skin samples of Foxn1-deficient nude mice vs Foxn1-active control mice) (Kur-Piotrowska *et al*.)^10^			Sequencing results (Kur-Piotrowska *et al*.)^10^			
			nude skin (inactive Foxn1)		nude epidermis (inactive Foxn1)	
Experimental group	Protein name	Protein symbol	up	down	up	down
Keratinocytes Ad-GFP	major vault protein isoform X1	Mvp			5.37	
	Msn protein	Msn			2.63	
	plastin 3	Pls3	1.39		2.37	
	keratin, type II cytoskeletal 7	Krt7			3.17	
	6-phosphogluconate dehydrogenase, decarboxylating	Pgd			2.63	
	PDZ and LIM domain protein 1	Pdlim1			2.34	
	annexin A1	Anxa1	4.51			
	annexin A4	Anxa4	3.97		1.34	
	Prohibitin	Phb			1.21	
	phosphoglycerate mutase 1	Pgam1			1.69	
Keratinocytes Ad-Foxn1	enoyl-CoA hydratase, mitochondrial precursor	Echs1		2.08		
	heat shock 70 kDa protein 1B	Hspa1a	4.48			1.86
	annexin A8	Anxa8				2.06
	voltage-dependent anion-selective channel protein 1	Vdac1		3.38		
			**Literature data**			
Keratinocytes Ad-Foxn1	1-phosphatidylinositol 4,5-bisphosphate phosphodiesterase delta-1	Plcd1	(Janes *et al*.)^23^			
	galectin-7	Lgals7	(Janes *et al*.)^23^			

### Foxn1 overexpression modulates the abundance of “hypoxic proteins”

**Figure 3:**
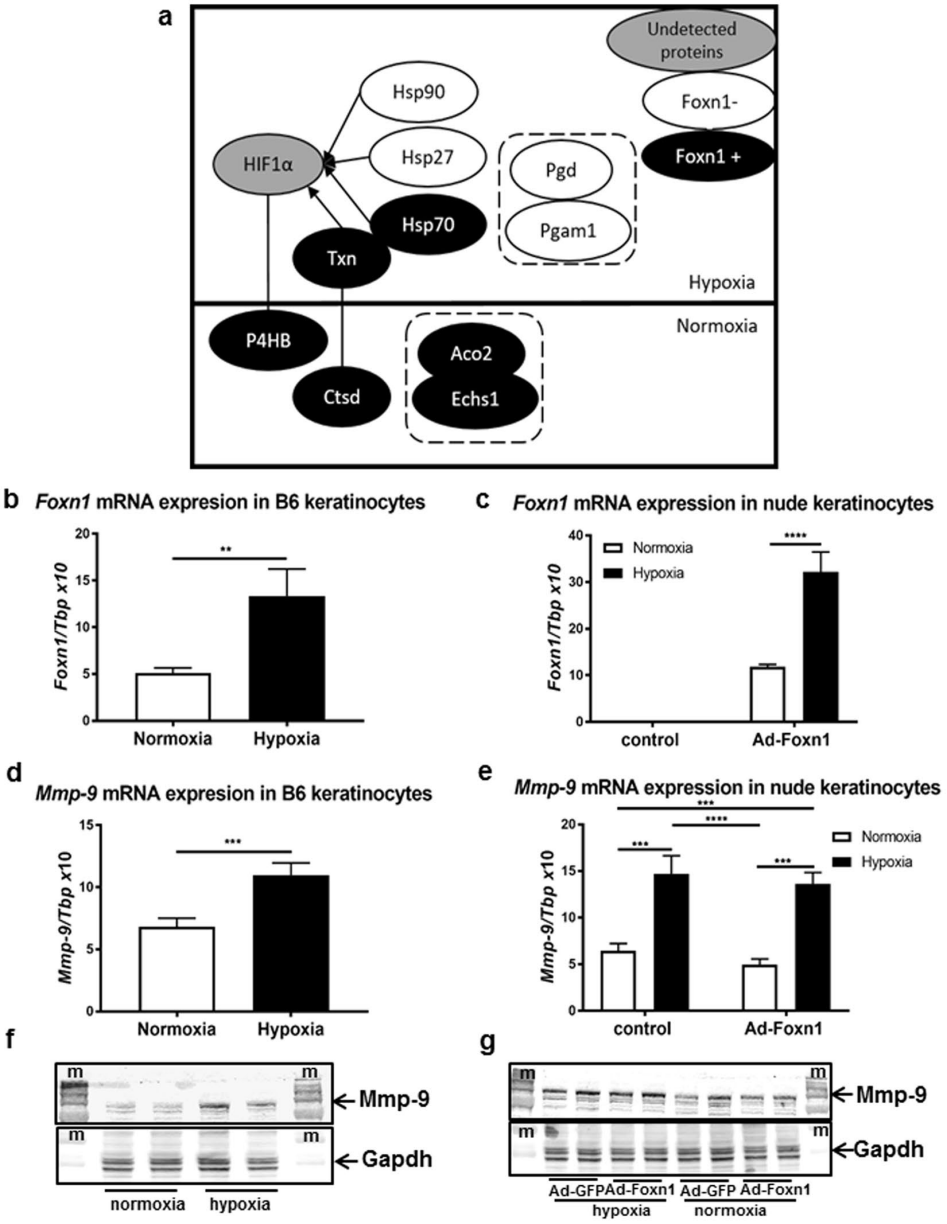
Foxn1 overexpression in keratinocytes affects proteins that regulate the hypoxia/normoxia response. Schematic representation of molecular cross-talk between proteins exhibiting hypoxic or normoxic characteristics, according to proteome profiling results, and induced in keratinocytes transfected with Ad-Foxn1 (black colour) or Ad-GFP (control; white colour) (**a**). Grey colour indicates proteins that were not detected in the analysis but link identified proteins (**a**). *Foxn1* mRNA expression in primary cultures of B6 keratinocytes (**b**) and Ad-Foxn1 or Ad-GFP (control)-transfected nude keratinocytes (**c**) cultured under normoxic (21% O_2_) or hypoxic (1% O_2_) conditions. Corresponding *Mmp-9* mRNA (**d**) and Mmp-9 protein (**f**) expression in keratinocytes isolated from B6 mice and *Mmp-9* mRNA (**e**) and Mmp-9 protein (**g**) expression in nude keratinocytes transfected with Ad-Foxn1 or Ad-GFP (control) cultured under normoxic (21% O_2_) or hypoxic (1% O_2_) conditions. Values are the mean ± SD; **p < 0.01, ***p < 0.001, ****p < 0.0001; m – molecular size marker.

A key aspect of wound closure is the migration of the keratinocytes that are affected by the hypoxic environment at the site of injury^25^. Hypoxia inducible factor 1 α (Hif1α), as a master regulator allowing cells to adapt to low oxygen concentrations, is constitutively produced in cells but rapidly degraded in the presence of oxygen^26^. Our proteomic data analysis pointed to proteins involved in Hif1α-/hypoxia-activated pathways (Fig. 3a). Foxn1 transfection into keratinocytes strongly (3.55-fold) induced thioredoxin (Txn, Table 1, see Supplementary Table S1), the main free radical scavenger that enhances Hif1α protein levels through Akt, p70S6 kinase (p70S6K) and eukaryotic initiation factor-4E (eIF-4E) activity^27,28^. Interestingly, Ad-Foxn1-transfected cells simultaneously revealed higher expression of enzymes with activity opposite to that of Txn: prolyl 4-hydroxylase (P4hb), the enzyme responsible for Hif1α degradation^29^, and cathepsin D (Ctsd) a protein involved in Txn degradation^30^ (Table 1, Fig. 3a). Ad-Foxn1-transfected keratinocytes showed elevated abundance of both P4hb and Ctsd (1.09- and 1.34-fold, respectively, compared with Ad-GFP-transfected keratinocytes; Table 1, see Supplementary Table S1). They also exhibited elevated levels of other “normoxic proteins”, for example, mitochondrial aconitase (Aco2) and enoyl-CoA hydratase (Echs1), which are enzymes depleted in “metabolic hypoxia” (Table 1, Fig. 3a, and see Supplementary Table S1)^31^. The data also showed the overproduction of heat shock protein 90 and 27 (Hsp90 and Hsp27) in control cells but underproduction of Hsp70 in keratinocytes transfected with Ad-Foxn1. Collectively, these results suggest that Foxn1 affects the abundance of proteins that allow cells to adapt to low-oxygen conditions. Interestingly, Foxn1 activity has opposing effects in the contexts of hypoxia and normoxia (Fig. 3a), underlining the importance of the sustained balance required for cell survival.

### Hypoxia induces Foxn1 expression

The number of Hif1α-/hypoxia-activated pathway-associated proteins detected in the proteomics analysis, together with the knowledge that hypoxia strongly influences early events after injury, impelled us to test how low oxygen concentration affects endogenous (B6 keratinocytes) and exogenous (nude mouse keratinocytes transfected with Ad-Foxn1) Foxn1 expression. We cultured monolayer keratinocytes isolated from B6 mice in normoxic (21% O_2_) or hypoxic (1% O_2_) conditions. qRT-PCR analysis of the collected samples revealed that *Foxn1* mRNA expression levels are significantly elevated under hypoxic conditions (Fig. 3b). Similarly, the induction of *Foxn1* mRNA has also been observed in B6 keratinocytes cultured with a chemical mimetic of hypoxia (DMOG; see Supplementary Figure S5A). These data strongly suggest that a hypoxic environment stimulates the expression of endogenous Foxn1. Next, using keratinocytes isolated from nude mice (Foxn1-deficient), we tested whether hypoxic conditions also stimulate exogenously introduced Foxn1. Indeed, Foxn1 was detected only in Ad-Foxn1-transfected cells, and *Foxn1* mRNA expression was significantly induced by hypoxia (Fig. 3c).

To our knowledge, this is the first report showing the stimulation of *Foxn1* by hypoxic conditions.

### Induction of Mmp-9 expression in hypoxic conditions

The critical effect of culture conditions (normoxic vs hypoxic) on Foxn1 expression turned our attention back towards the initial research focus, that is, exploring the effect of Foxn1 on Mmp-9 expression (compare Fig. 1). We assumed that the initially obtained data were ambiguous due to conditions that did not support a physiological (hypoxic) environment during the wound healing process. Therefore, the samples collected in the experiments described above (hypoxia vs normoxia) were analysed further with qRT-PCR and Western blot for Mmp-9 mRNA and protein expression. Indeed, B6 keratinocytes displayed elevated levels of *Mmp-9* mRNA expression (p < 0.01) accompanied by an increase in *Foxn1* mRNA expression under hypoxic conditions (compare Fig. 3b and d). Western blot analysis confirmed the increase in Mmp-9 protein content under hypoxic conditions (Fig. 3f). An increase in Mmp-9 expression under low-oxygen conditions was previously detected in cultured cell lines^32^ and human keratinocytes^33^. Here, we propose that Mmp-9 expression in Foxn1-positive keratinocytes is stimulated in a biphasic manner: hypoxia stimulates Foxn1, which in turn stimulates Mmp-9 expression. A study by O’Toole *et al*. revealed that in human keratinocytes, Mmp-9 is induced via the PKC pathway under hypoxic conditions^33^. Foxn1 antagonizes the effect of PKC, as was shown by Li *et al*.^19^ and as confirmed by our data (see Fig. 1j). Therefore, we suggest that there are other pathways through which Foxn1 can stimulate Mmp-9 activity. For example, Wnt11 has been shown to be elevated in hypoxic conditions, and it increases the activity of Mmp-9^32^; moreover, Wnt glycoproteins have been previously shown to regulate Foxn1 expression in the thymus^34^. Thus, the potential mechanisms linking Foxn1 and Mmp-9 may engage the Wnt pathway.

The ability of low-oxygen conditions to induce Mmp-9 expression was further examined in the nude mouse keratinocyte model (Fig. 3e and g). Mmp-9 mRNA and protein expression levels were significantly elevated in nude keratinocytes under hypoxic conditions (Fig. 3e and g). However, the hypoxia-stimulated increase in Mmp-9 expression was detected in both the presence and absence of Foxn1 expression (i.e., in both Ad-Foxn1- and Ad-GFP-transfected keratinocytes) (Fig. 3e and g). Similar levels of Mmp-9 mRNA (Fig. 3e) and protein (Fig. 3g) expression were detected in Ad-Foxn1- and Ad-GFP-transfected (control) keratinocytes. Interestingly, the hypoxia-stimulated increase in endogenous *Foxn1* expression detected in B6 keratinocytes (Fig. 3b) strongly corresponds with the Mmp-9 up-regulation under hypoxic conditions (Fig. 3d and f), but exogenous/forced Foxn1 expression in nude keratinocytes under hypoxic conditions (Fig. 3c) induced comparable levels of *Mmp-9* expression in both Foxn1-transfected and control (Ad-GFP transfected) keratinocytes (Fig. 3e and g).

One of the possible explanations for these observations comes from our previous study^10^. Using next-generation high-throughput DNA sequencing techniques, we showed that Foxn1 appeared to be an essential element for establishing the adult skin phenotype; the lack of Foxn1 activity interferes with the developmental programme of keratinocytes and total skin maturation, and this is the apparent cause of skin immaturity in nude mouse^10^. The present data seems to confirm our previous findings, showing that immaturity of nude keratinocytes caused by a lack of Foxn1 expression during development results in the inability of nude keratinocytes to respond to forced Foxn1 introduction. Detected in low-oxygen conditions the comparable increase of Mmp-9 expression in nude keratinocytes transfected with either Ad-Foxn1 or Ad-GFP suggests other than Foxn1 related mechanisms.

### Foxn1 overexpression in keratinocytes regulates the abundance of pro-survival and pro-apoptotic proteins

Local stress caused by skin injury results in a variety of cell responses, ranging from the activation of pro-survival pathways to the activation of programmed cell death^35,36^. Our proteomics analysis of the differently expressed proteins revealed that the proteins that are up-regulated as a result of Foxn1 activity are enriched for pro-survival and pro-apoptotic functions.

**Figure 4:**
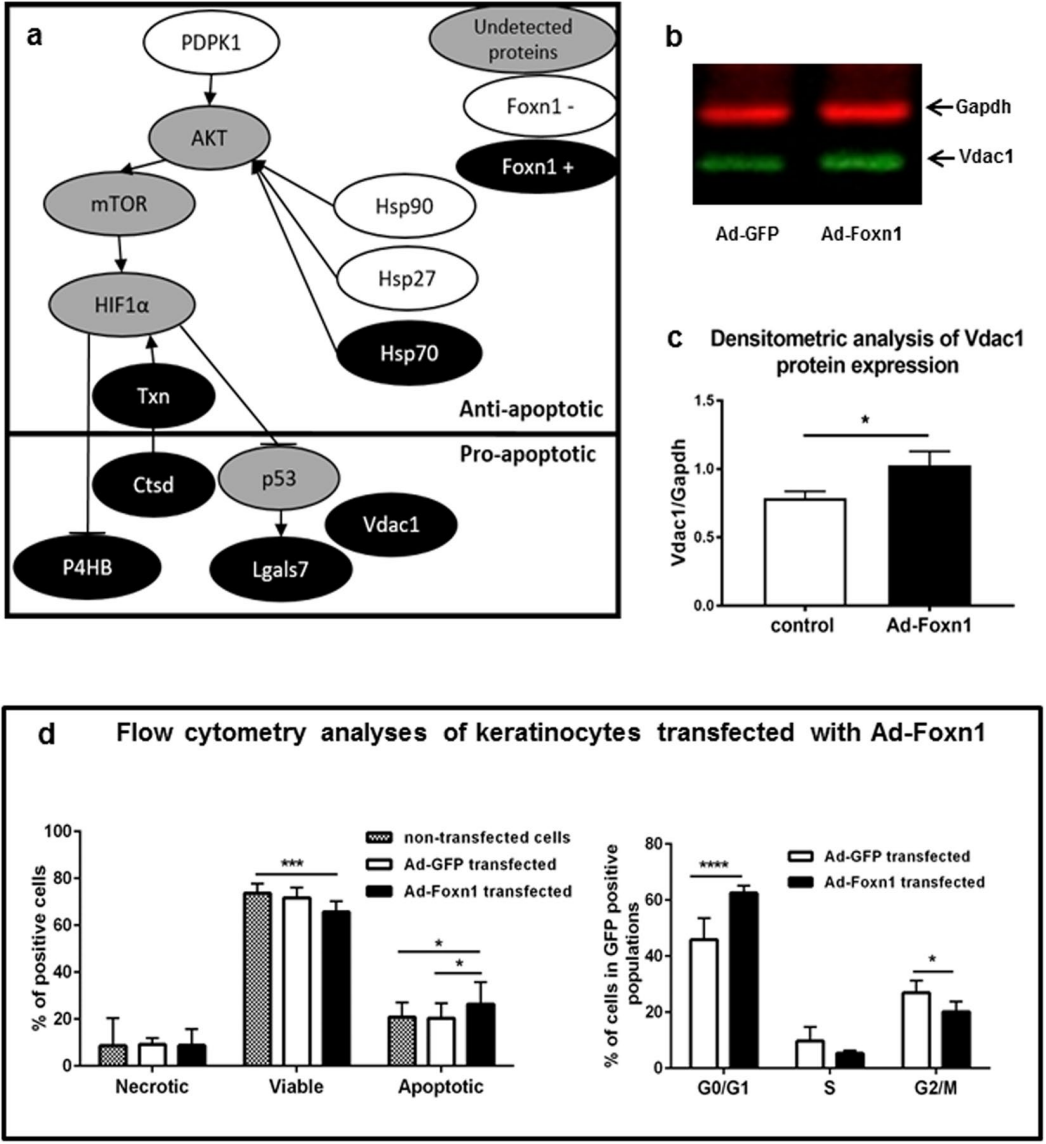
Foxn1 overexpression in keratinocytes affects pro-survival and pro-apoptotic protein profiles. Schematic representation of molecular cross-talk between proteins exhibiting pro- or anti-apoptotic characteristics, according to proteome profiling results, and induced in keratinocytes transfected with Ad-Foxn1 (black colour) or Ad-GFP controls (white colour) (**a**). Grey colour indicates proteins that were not detected in the analysis but link identified proteins (**a**). Representative Western blot (**b**) and densitometric analysis (**c**) (n = 4) of Vdac1 protein expression in keratinocytes transfected with Ad-GFP (control) or Ad-Foxn1. Full-length blots are presented in Supplementary Fig. S6. Flow cytometric analysis of keratinocytes transfected with Ad-Foxn1 or Ad-GFP (control) showing the percentages of necrotic, viable and apoptotic cells and cells in G0/G1, S or G2/M phase (**d**). The values are the mean ± SD; *p < 0.05, ***p < 0.001; ****p < 0.0001.

The most prominent change was detected in Pdpk1, which was up-regulated up to 8.21-fold in control keratinocytes (Table 1, and see Supplementary Table S1). Pdpk1, acting upstream of Akt (protein kinase B), is involved in the pro-proliferative pathway^37,38^ (Fig. 4a). Moreover, Hsp90 and Hsp27, which are essential for cell survival in stress conditions^35^, were more abundant in control keratinocytes (Table 1, Fig. 4a, and see Supplementary Table S1). Another heat shock protein, Hsp70, exhibited similar pro-survival effects^39^, but in contrast to Hsp90 and Hsp27, Hsp70 is induced (1.86-fold) in cells overexpressing Foxn1 (Table 1, and see Supplementary Table S1).

In line with our observation that control keratinocytes are characterized by the presence of pro-survival proteins, some of the proteins detected in Foxn1-overexpressing keratinocytes exhibit pro-apoptotic characteristics, for example, P4hb^40^, Lgals7 and voltage-dependent anion-selective channel protein 1 (Vdac1) (Table 1, Fig. 4a, and see Supplementary Table S1). Lgals7 has been shown to be a target of p53, a pro-apoptotic molecule that is dysregulated in many cancers^41,42^. Vdac1, a mitochondrial outer membrane protein, has been characterized as a gatekeeper that controls cell metabolism and may induce the release of cytochrome C and apoptosis through its oligomerization^43^. Western blot analysis for Vdac1 expression showed significantly higher levels of Vdac1 protein in Foxn1-overexpressing keratinocytes, confirming our proteomics data (Fig. 4b,c, and see Supplementary Figure S6).

Next, we tested whether Foxn1-overexpressing keratinocytes, as well as exhibiting elevated levels of pro-apoptotic proteins, are actually predisposed to programmed cell death. Flow cytometric analysis of keratinocytes transfected with Ad-Foxn1 revealed an increase in the population of apoptotic cells in Foxn1-overexpressing keratinocytes relative to the Ad-GFP-transfected (p < 0.05) and non-transfected (p < 0.05) cells (Fig. 4d, and see Supplementary Figure S7). Simultaneously, decreased viability was detected in Ad-Foxn1-transfected keratinocytes (Fig. 4d; p < 0.001 vs non-transfected cells). Flow cytometry analysis showed an increase in the apoptotic population in Ad-Foxn1 keratinocytes, supporting the proteomics (Table 1, Fig. 4a, and see Supplementary Table S1) and Western blot (Fig. 4b and c) data. Moreover, flow cytometry showed that cells transfected with Ad-Foxn1 contain a significantly higher percentage of keratinocytes in G0/G1 phase and a significantly lower percentage in G2/M phase compared to the control cell cycle distribution (Fig. 4d, and see Supplementary Figure S7). The accumulation of the majority of Ad-Foxn1-transfected cells in G0/G1 phase may suggest the dominance of the non-proliferating state in this population of cells, which is additionally supported by the increase in the proportion of K10-positive (i.e., differentiated) cells among the Ad-Foxn1-transfected keratinocytes (see Fig. 2a).

Foxn1 seems to act as a double-edged sword in terms of cell fate. In Ad-Foxn1-transfected keratinocytes, we detected both pro- and anti-apoptotic proteins, the balance of which appears to dictate the choice between proliferation and the activation of the pathways that finally lead to death. This observation is in accordance with reports showing Foxn1 as a master regulator promoting terminal differentiation and controlling the steps in the differentiation programme^19,23,44,45^. Interestingly, similar conclusions concerning the abundance of pro- and anti-apoptotic proteins have been obtained in the proteomic analysis of regenerating urodele limbs^46^. Together, these observations indicating the possible role of Foxn1 in pro-apoptotic pathways further support the hypothesis that a lack of Foxn1 activity may favour regenerative skin wound healing^10^.

## Conclusion

Our long-term investigation aims to dissect the role of the transcription factor Foxn1 in reparative, scar-forming skin wound healing in order to identify the pathways whose modulation may activate the regenerative pathways detected in Foxn1-deficient (nude) mice. The present study showed that Foxn1 allows cells to adapt to hypoxic conditions and can act as a factor controlling the balance between cell proliferation, differentiation and apoptosis. Under low-oxygen conditions, Foxn1 expression is induced in primary cultures of keratinocytes. We also showed that hypoxia-stimulated Foxn1 expression in B6 keratinocytes is coincident with an increase in Mmp-9 expression. Further study is required to determine whether there is a direct stimulatory link between Foxn1 and Mmp-9.

## Materials and Methods

### Animals

The study was performed on C57BL/6 (B6), Foxn1::Egfp transgenic, nude (Cby.Cg-Foxn1<nu>/cmdb) and BALB/c (BALB/c/cmdb) mice. Keratinocytes and dermal fibroblasts (DFs) for *in vitro* studies were always isolated from newborn mice. *In vivo* studies were carried out on young (21- to 28-day-old) Foxn1::Egfp transgenic mice.

All animals were bred and housed in a temperature- and humidity-controlled room (22 ± 2 °C and 30–70% humidity) with a 12-h light/12-h dark cycle at the Institute of Animal Reproduction and Food Research, Polish Academy of Sciences, Olsztyn, Poland (B6, Foxn1::Egfp transgenic mice) or the Center of Experimental Medicine, Medical University of Bialystok, Poland (nude mice).

All experimental animal procedures were approved by the Ethics Committee of the University of Warmia and Mazury (Olsztyn, Poland), No. 28/2012. The study was carried out in accordance with EU Directive 2010/63/EU of the European Parliament and of the Council on the Protection of Animals Used for Scientific Purposes (OJEU, 2010. Official Journal of the European Union. Directive 2010/63/EU of the European Parliament and of the Council on the protection of animals used for scientific purposes. OJEU. [cited 2010 Oct 20]; Series L 276:33–79).

### Cell Culture

Mouse keratinocytes and DFs were isolated from newborn B6, BALB/c and nude mice. Skin tissues were incubated in dispase (6 U/ml; Life Technologies) overnight at 4 °C. Then, separated epidermis was digested in 0.05% trypsin-EDTA (Life Technologies) for 3 min and filtered through a 70 µm strainer. Next, keratinocytes were collected by a series of three rounds of trypsin digestion (at 37 °C) and filtration followed by centrifugation at 300 × *g* for 9 min at room temperature. The pelleted cells were suspended and seeded in Dulbecco’s modified Eagle’s medium (DMEM/F-12; Sigma-Aldrich) supplemented with 10% foetal bovine serum (FBS; Life Technologies), 0.2% Primocin (InvivoGen), and 120 µM β-mercaptoethanol (Sigma-Aldrich). The media were exchanged for CnT medium (CELLnTEC) 24 h after seeding.

The remaining dermal tissues were digested for 45 min in collagenase type I (220 U/ml; Life Technologies), filtered through a 70 μm strainer and centrifuged. Isolated DFs were seeded in DMEM/F-12 medium containing 15% FBS and antibiotics (Penicillin/Streptomycin, Sigma-Aldrich). All *in vitro* experiments were performed on primary keratinocytes and DFs (p = 0).

### Keratinocyte transfection

Foxn1-GFP (MG226744) and Foxn1-Ddk (MR226744) plasmid vectors were purchased from OriGene. Control plasmids were obtained by digestion of Foxn1-GFP and Foxn1-Ddk with the restriction enzymes SgfI and MluI (Promega) to excise Foxn1 cDNA. Fragments whose molecular size corresponded to linear DNA without the Foxn1 sequence were isolated from agarose gels using Gel-Out Concentrator (A&A Biotechnology). Samples were concentrated with DNA Clean & Concentrator (Zymo Research). Linear DNA without Foxn1 sequence was ligated with T4 DNA ligase (A&A Biotechnology). All plasmids were propagated in *E. coli*. Solutions containing 2 µg of plasmid DNA/well (or 1 µg/insert) and Lipofectamine LTX & Plus Reagent (Life Technologies) in 0.5 ml of CnT Basal Medium (CELLnTEC) were prepared according to the manufacturer’s protocol for plasmid transfection.

The Foxn1-GFP-expressing (Ad-Foxn1) and control (Ad-GFP) adenovirus was a kind gift from Janice L. Brissette (Harvard Medical School, Boston, MA)^19^. Large-scale adenovirus preparation was performed at Baylor College of Medicine, Houston, TX, USA; by Jakub Siednienko at the Institute of Immunology and Experimental Therapy, Polish Academy of Sciences, Wroclaw, Poland, and by Artur Padzik at the Turku Centre for Biotechnology, Turku, Finland. Adenoviral infections were performed at 200 MOI in 0.5 ml of CnT Basal Medium (CELLnTEC).

Keratinocytes at 70% confluence were transfected with plasmid or adenovirus. After 4 hours, 1 ml of CnT Basal Medium with supplements A, B, and C (CELLnTEC) was added per insert/well. In experiments performed under hypoxic conditions, cells were cultured in a humidified incubator with 1% O_2_ (37 °C) for 24 h. For co-culture experiments, keratinocytes (cultured in inserts) and DFs (cultured on the bottoms of 6-well plates) were set up together 24 h after keratinocyte transfection and cultured for 48 h.

For proteomic analysis, after 24 h of transfection, media were exchanged for CnT Basal Medium (without supplements) for cultured keratinocytes and DMEM/F-12 (without FBS) for cultured DFs. Subsequently, co-culture systems consisting of keratinocytes transfected with Ad-Foxn1 or Ad-GFP and DFs were established and cultured for another 48 h.

### *In vitro* wound migration assay

For the wound migration assay, keratinocytes at passage 0 (p = 0) were plated in 12-well plates at a density of 0.5 × 10^6^ per well for 48 h, at which point cells reached subconfluence. Then, cells were transfected with Ad-Foxn1 or Ad-GFP for 4 h. Non-transfected keratinocytes were used as a control. To prevent cell proliferation, keratinocytes were cultured for 24 h and then incubated for another 3 h with mitomycin C (10 µg/ml)^15^ in CnT Basal Medium supplemented with 0.2% FBS. Then, cell monolayers were wounded by scratching in a straight line throughout the centre of the entire 12-well plate with a 200-µl pipet tip. Debris were removed by washing the cells with PBS. All treatments were performed in duplicate, and each experiment was repeated two times using cells isolated from 11 animals. Images were captured with an Olympus microscope (IX51) equipped with an Olympus digital camera (XC50) and analysed with ImageJ (SciJava software, National Institutes of Health; NIH). Three representative images of scratched areas were photographed, and the distance between the unclosed edges was measured. The distance of scratch closure at 0 h was considered to be 100%. The scratched areas were monitored until closure (at 0, 20, 32, 44, 56 and 68 h).

### Proteomics

#### Sample Preparation

Keratinocytes transfected with Ad-Foxn1 or Ad-GFP and co-cultured for 48 h with DFs (n = 4, 2 animals per sample; total n = 8 animals) were collected in DIGE buffer (30 mM Tris, 7 M urea, 2 M thiourea, and 4% CHAPS (3-[(3-cholamidopropyl) dimethylammonio]-1-propanesulfonate hydrate)) and sonicated (Sonics Vibro-Cell ultrasound sonicator (3 × 20 sec, 20 kHz). After centrifugation (10000 × *g* for 15 min at 4 °C), supernatants were precipitated using the 2D Clean-up Kit (GE Healthcare), and pellets were resuspended in DIGE labelling buffer.

#### 2D-DIGE analysis of Ad-Foxn1 and Ad-GFP keratinocytes

Protein labelling with CyDye DIGE Fluor and 2D electrophoresis were performed as previously described by Dietrich *et al*.^47^. Briefly, 50 μg of each sample were minimally labelled by incubation with 400  pmol of amine-reactive cyanine dye (Cy3 or Cy5) for 30 min. The internal standard was generated by combining equal amounts of proteins from each of 8 samples and labelling with Cy2. A dye swap (Cy3/Cy5) was performed between Ad-Foxn1 and Ad-GFP samples (see Supplementary Table S2) to exclude dye bias. Differentially labelled samples were mixed together (see Supplementary Table S2). In each gel, therefore, extract from Ad-Foxn1 (Cy3 or Cy5) and Ad-GFP (Cy5 or Cy3) and internal standard (Cy2) were separated. The samples were loaded onto Immobiline DryStrip gel strips (18 cm, pH 3 to 10 non-linear; GE Healthcare). Isoelectric focusing was performed with an IPGphor isoelectric focusing unit (GE Healthcare), and SDS-PAGE was run using the ETTAN Dalt six electrophoresis unit (GE Healthcare) as described by Nynca *et al*. 2015^48^.

#### Image Acquisition and Quantitative Analysis

The CyDye-labelled gels were analysed by post-run fluorescence imaging with the use of a Typhoon FLA 9500 instrument (GE Healthcare). After the multiplexed images were acquired, image analysis was performed with the use of DeCyder Differential Analysis Software (version 5.0; GE Healthcare). Differential in-gel analysis was used to calculate protein abundance variations between samples on the same gel. The resulting spot maps were then analysed by biological variation analysis to provide statistical data on the differential protein expression between Ad-Foxn1 and Ad-GFP keratinocyte proteomes. Changes in protein abundance were considered to be relevant if (1) the corresponding spots were detected in all gels, and (2) Student’s t test reached levels of significance, with P ≤ 0.05 (including false discovery rate correction). After analysis, gels were stained with Coomassie Brilliant Blue R-250 (Bio-Rad). Spots presenting significant differences between Ad-Foxn1 and Ad-GFP keratinocyte proteomes were manually excised, trypsin digested, and identified with matrix-assisted laser desorption/ionization time-of-flight/time-of-flight mass spectrometry (MALDI TOF/TOF, Bruker Daltonics, Bremen, Germany).

#### MALDI TOF/TOF Protein Identification

Spots of interest were cut from the gel and prepared for identification as previously described by Slowinska *et al*.^49^. MS peptide mass fingerprint and fragment spectra from each individual spot were combined and used to search against the NCBIr *Mus musculus* nr_11.09.2017 database using Mascot (version 2.4; Matrix Science Ltd, London, UK) with the following criteria: enzyme, trypsin; fixed modification, carbamidomethylation (C); variable modifications, oxidation (M); peptide mass tolerance, 200 ppm; fragment mass tolerance, 0.7 Da; missed cleavages allowed, 1. The search results were filtered with a significant threshold of p < 0.05 and a Mascot ion score cut-off of ≥30 for at least two peptides.

The mass spectrometry proteomics data have been deposited with the ProteomeXchange Consortium via the PRIDE^50^ partner repository with the dataset identifier PXD007843.

### Real-time RT-qPCR

Real-time RT-qPCR was performed as previously described^9^. Briefly, total RNA was extracted from cell culture using TRI Reagent (Sigma-Aldrich). cDNA was synthesized from 500 ng of total RNA using High-Capacity cDNA Reverse Transcription Kit with RNase Inhibitor (Applied Biosystems). mRNA levels of *Foxn1*, *Mmp-9*, hypoxanthine phosphoribosyltransferase 1 (*Hprt1*), and TATA-binding protein (*Tbp*) were measured with Single-Tube TaqMan® Gene Expression Assays (Life Technologies). The levels of gene expression were quantified relative to the level of *Hprt1* or *Tbp* using the standard curve method.

### Protein extraction and Western blot

Western blot was performed as described^8^. Briefly, total cell lysates were prepared in 100–400 µl RIPA buffer containing protease inhibitor cocktail (PhosStop, Roche; Protease Inhibitor, Sigma). The blots were incubated with anti-Mmp-9 (1:750, rabbit anti-mouse, AB19016, Millipore), anti-cytokeratin 16 (1:500, rabbit anti-mouse, NB100-91842, Novus Biologicals), anti-involucrin (1:1000, rabbit anti-mouse, PRB-140C-200, Covance), anti-Vdac1 (1:250, sc-32063, Santa Cruz), anti-phospho-PKC (pan) (1:1000, 9371, Cell Signaling Technology), or anti-Gapdh (1:2000, mAbcam 9484, AbCam) primary antibodies and subsequently with fluorescent IRDye 800 (1:1000, goat anti-rabbit, 611-132-122, Rockland), IRDye 800 (1:1000, donkey anti-goat, 925–32214, LI-COR Biotechnology), Alexa Fluor 680 (1:1000, donkey anti-mouse, A10038, Life Technologies) or Cy 5.5 (1:1000, goat anti-mouse, 610-113-121, Rockland) secondary antibodies. Bands were visualized using the Odyssey imaging system (LI-COR Bioscience) according to the manufacturer’s protocol. The samples (n = 3–4) were loaded on one blot and analysed (each sample comprised cultured cells isolated from 6–8 newborn mice). Densitometric protein analysis for Vdac1 was performed with ImageJ software, available at http://rsb.info.nih.gov/ij/index.html.

### Flow cytometry analysis

Non-transfected keratinocytes and keratinocytes transfected with Ad-Foxn1 or Ad-GFP were analysed for K10 and K14 expression using flow cytometry methods. Keratinocytes were trypsinized 48 hours after transfection, suspended in warm PBS and incubated for 30 min with the following antibodies: anti-keratin 10-PerCP (mouse monoclonal, clone SPM261; Novus Biologicals conjugated with PerCP; Lynx Rapid PerCP antibody conjugation kit; AbD Serotec) and anti-keratin 14-APC (mouse monoclonal, clone LL002; Novus Biologicals conjugated with APC; Lynx Rapid APC antibody conjugation kit; AbD Serotec). To determine the percentage of viable, apoptotic and necrotic cells, keratinocytes collected by trypsinization (0.05% trypsin-EDTA) were resuspended in Annexin Binding Buffer (Life Technologies). Then, Annexin V Alexa Fluor 350 Conjugate (Life Technologies) and propidium iodide (Life Technologies) were added. For characterization of the cell cycle phase distribution, a BD Cycletest Plus DNA Kit (BD Biosciences) was used according to the manufacturer’s description. Samples (n = 4 for keratin analysis, n = 8 for cell viability and n = 7 for cell cycle phase) were analysed using flow cytometry with BD LSR Fortessa Cytometer running Diva software 6.2 (BD Biosciences).

### Wound model and immunofluorescence

Skin samples from injured skin of Foxn1::Egfp transgenic mice were collected as described^8^. Samples were fixed for 2 h in 4% paraformaldehyde (Sigma-Aldrich). Mmp-9 detection was performed on cryostat sections with Mmp-9 primary antibody (1:100, rabbit anti-mouse, Millipore), followed by secondary antibody Alexa Fluor 594 (1:200, goat anti-rabbit, Life Technologies) as^8^. Sections were visualized and photographed with an Olympus microscope (BX43) equipped with an Olympus digital camera (XC50).

### Statistical analysis

Quantitative RT-PCR, flow cytometry and Western blot densitometric data were analysed using GraphPad Prism, version 6.0 (GraphPad Software Inc., CA, USA). The means and SEM were calculated for each dataset. One-way analysis of variance (ANOVA) with Tukey’s multiple comparisons test and Student’s t test were used for analysing differences between experimental groups as indicated in the figure legends. A value of p < 0.05 was considered significant.

## Electronic supplementary material

Supplementary Dataset 1
