# Supplementary material for: Foxn1 expression in keratinocytes is stimulated by hypoxia: further evidence of its role in skin wound healing
Source: Sci Rep. 2018 Apr 3;8:5425. doi: 10.1038/s41598-018-23794-5 (PMC5882803; doi:10.1038/s41598-018-23794-5)

**Supplementary Data**

**Foxn1 expression in keratinocytes is stimulated by hypoxia: further evidence of its role in skin wound healing process**

Anna Kur-Piotrowska, Joanna Bukowska, Marta M. Kopcewicz, Mariola Dietrich, Joanna Nynca, Mariola Slowinska and Barbara Gawronska-Kozak^*^

**Supplementary Tables:**

Supplementary Table 1 pg 2-4

Supplementary Table 2 pg 5

Supplementary Table 3 pg 6-7

**Supplementary Figures:**

Supplementary Figure 1 pg 8-9

Supplementary Figure 2 pg 10

Supplementary Figure 3 pg 11

Supplementary Figure 4 pg 12

Supplementary Figure 5 pg 13

Supplementary Figure 6 pg 14

Supplementary Figure 7 pg 15

**Table S1**

Proteins identified as differentially abundant in keratinocytes transfected with Ad-Foxn1 compared to control keratinocytes (transfected with Ad-GFP). Results highlighted in grey color correspond to proteins more abundant in keratinocytes overexpressing Foxn1 relative to the control.

| **Differentially abundant proteins in keratinocytes transfected with Ad-Foxn1/Ad-GFP** | | | | | | | | |
| --- | --- | --- | --- | --- | --- | --- | --- | --- |
| Pos. | Protein name *(organism)* | Gene symbol | GI number | Protein score | Sequence coverage | No peptides (ion score >30) | MW/pI | Av. Ratio |
| 1 | PREDICTED: major vault protein isoform X1 *(Mus musculus)* | Mvp | XP_011240254.1 | 856 | 44 | 6 | 96.15/5.43 | 1.18 |
| 2 | PREDICTED: alpha-actinin-4 isoform X4 (*Mus musculus)* | Actn4 | XP_006540320.1 | 814 | 48 | 9 | 107.75/5.23 | 1.20 |
| 3 | PREDICTED: aconitate hydratase, mitochondrial *(Mesocricetus auratus)* | Aco2 | XP_012966696.1 | 314 | 18 | 4 | 85.77/7.87 | 1.15 |
| 4 | mitochondrial aconitase *(Rattus norvegicus)* | Aco2 | CAC11018.1 | 444 | 19 | 5 | 86.16/7.87 | 1.24 |
| 5 | gelsolin isoform 2 *(Mus musculus)* | Gsn | NP_001193296.1 | 770 | 33 | 7 | 80.98/5.52 | 1.21 |
| 6 | PREDICTED: caldesmon isoform X11 *(Mus musculus)* | Cald1 |  | 284 | 27 | 4 | 61.86/6.60 | 1.48 |
| 7 | caldesmon *(Mus musculus)* | Cald1 | NP_663550.1 | 369 | 34 | 3 | 60.53/6.97 | 1.24 |
| 8 | not identified |  |  |  |  |  |  | 1.40 |
| 9 | not identified |  |  |  |  |  |  | 2.16 |
| 10 | PREDICTED: 1-phosphatidylinositol 4,5-bisphosphate phosphodiesterase delta-1 isoform X1 *(Mus musculus)* | Plcd1 | XP_006512047.1 | 282 | 27 | 2 | 79.70/5.67 | 1.33 |
| 11 | moesin protein, partial  *(Mus musculus)* | Msn | AAH03474.1 | 351 | 43 | 3 | 51.99/8.84 | 1.35 |
| 12 | 3-phosphoinositide-dependent protein kinase 1 isoform 1 *(Homo sapiens)* | Pdpk1 | NP_002604.1 | 344 | 43 | 5 | 63.62/6.95 | 8.21 |
| 13 | 3-phosphoinositide dependent protein kinase-1, partial *(synthetic construct)* | Pdpk1 | AAX29847.1 | 657 | 54 | 8 | 63.73/6.95 | 4.12 |
| 14 | 3-phosphoinositide-dependent protein kinase 1 isoform 1 *(Homo sapiens)* | Pdpk1 | NP_002604.1 | 388 | 31 | 5 | 63.62/6.95 | 3.36 |
| 15 | 3-phosphoinositide dependent protein kinase-1 variant, partial *(Homo sapiens)* | Pdpk1 | BAD96301.1 | 241 | 22 | 2 | 63.59/6.95 | 3.48 |
| 16 | 3-phosphoinositide-dependent protein kinase 1 isoform 1 *(Homo sapiens)* | Pdpk1 | NP_002604.1 | 781 | 48 | 10 | 63.62/6.95 | 6.18 |
| 17 | heat shock 70 kDa protein 1B *(Mus musculus)* | Hspa1a | NP_034608.2 | 764 | 43 | 8 | 70.42/5.53 | 1.86 |
| 18 | plastin 3 (T-isoform), isoform CRA_b, partial *(Mus musculus)* | Pls3 | EDL29217.1 | 1020 | 44 | 10 | 77.59/5.71 | 1.28 |
| 19 | PREDICTED: plastin-3 isoform X1 *(Mus musculus)* | Pls3 | XP_006527794.1 | 385 | 40 | 6 | 72.73/5.42 | 1.26 |
| 20 | PREDICTED: plastin-3 isoform X1 *(Mus musculus)* | Pls3 | XP_006527794.1 | 914 | 51 | 10 | 72.73/5.42 | 1.45 |
| **Cont., Table S1** | | | | | | | | |
| 21 | type II keratin 5 *(Mus musculus)* | Krt5 | AAL16774.1 | 437 | 38 | 4 | 61.97/7.59 | 1.32 |
| 22 | type II keratin 5 *(Mus musculus)* | Krt5 | AAL16774.1 | 717 | 44 | 7 | 61.97/7.59 | 1.35 |
| 23 | keratin, type II cytoskeletal 6A *(Mus musculus)* | Krt6a | NP_032502.3 | 355 | 30 | 4 | 59.64/8.04 | 1.25 |
| 24 | prolyl 4-hydroxylase, beta polypeptide, isoform CRA_a *(Mus musculus)* | P4hb | EDL34765.1 | 441 | 36 | 3 | 59.44/4.78 | 1.09 |
| 25 | keratin, type II cytoskeletal 6A *(Mus musculus)* | Krt6a | NP_032502.3 | 559 | 42 | 6 | 59.64/8.04 | 1.35 |
| 26 | type II keratin 5 *(Mus musculus)* | Krt5 | AAL16774.1 | 709 | 45 | 8 | 61.97/7.59 | 1.41 |
| 27 | keratin, type II cytoskeletal 6A *(Mus musculus)* | Krt6a | NP_032502.3 | 458 | 40 | 5 | 59.64/8.04 | 1.33 |
| 28 | Krt6b protein *(Mus musculus)* | Krt6b | AAI39808.1 | 467 | 44 | 5 | 60.57/8.33 | 1.35 |
| 29 | keratin, type II cytoskeletal 6A *(Mus musculus)* | Krt6b | NP_032502.3 | 560 | 54 | 6 | 59.64/8.04 | 1.21 |
| 30 | keratin 5 *(Mus musculus)* | Krt5 | EDL04029.1 | 496 | 63 | 5 | 61.95/7.59 | 1.38 |
| 31 | mCG17580 *(Mus musculus)* | Krt6a | EDL04030.1 | 681 | 56 | 5 | 59.64/8.04 | 1.34 |
| 32 | D-3-phosphoglycerate dehydrogenase *(Mus musculus)* | Phgdh | NP_058662.2 | 222 | 25 | 3 | 57.35/6.12 | 1.22 |
| 33 | keratin, type II cytoskeletal 6A *(Mus musculus)* | Krt6b | NP_032502.3 | 760 | 52 | 6 | 59.64/8.04 | 1.27 |
| 34 | keratin, type II cytoskeletal 7 *(Mus musculus)* | Krt7 | NP_149064.1 | 532 | 70 | 4 | 50.68/5.67 | 1.37 |
| 35 | perilipin-3 *(Mus musculus)* | Plin3 | NP_080112.1 | 421 | 45 | 4 | 47.35/5.45 | 1.14 |
| 36 | heterogeneous nuclear ribonucleoprotein H1, isoform CRA_d *(Mus musculus)* |  | EDL33701.1 | 127 | 38 | 1 | 49.54/5.89 | 1.18 |
| 37 | 6-phosphogluconate dehydrogenase, decarboxylating *(Mus musculus)* | Pgd | NP_001074743.1 | 248 | 27 | 2 | 53.73/6.81 | 1.23 |
| 38 | 6-phosphogluconate dehydrogenase, decarboxylating  *(Mus musculus)* | Pgd | NP_001074743.1 | 713 | 36 | 8 | 53.73/6.81 | 1.22 |
| 39 | plasminogen activator inhibitor 2, macrophage *(Mus musculus)* | Serpinb2 | NP_035241.1 | 1130 | 60 | 10 | 46.38/5.04 | 1.38 |
| 40 | cathepsin D precursor *(Mus musculus)* | Ctsd | NP_034113.1 | 427 | 34 | 6 | 45.38/6.71 | 1.34 |
| 41 | plasminogen activator inhibitor 2, macrophage *(Mus musculus)* | Serpinb2 | NP_035241.1 | 1070 | 63 | 8 | 46.38/5.04 | 1.46 |
| 42 | 26S proteasome non-ATPase regulatory subunit 13 *(Mus musculus)* | Psmd13 | NP_036005.1 | 354 | 44 | 4 | 43.12/5.46 | 1.22 |
| 43 | PDZ and LIM domain protein 1 *(Mus musculus)* | Pdlim1 | NP_058557.2 | 515 | 58 | 6 | 36.21/6.38 | 1.12 |
| 44 | arginase-1 *(Mus musculus)* | Arg1 | NP_031508.1 | 174 | 36 | 2 | 34.96/6.51 | 1.85 |
| 45 | arginase-1 *(Mus musculus)* | Arg1 | NP_031508.1 | 803 | 70 | 8 | 34.96/6.51 | 1.84 |
| 46 | PREDICTED: poly(rC)-binding protein 1 *(Rattus norvegicus)* | Pcbp1 | XP_008774006.1 | 388 | 58 | 4 | 36.00/7.03 | 1.26 |
| 47 | arginase-1 *(Mus musculus)* | Arg1 | NP_031508.1 | 740 | 62 | 10 | 34.96/6.51 | 1.52 |
| **Cont., Table S1** | | | | | | | | |
| 48 | PREDICTED: LIM and SH3 domain protein 1 isoform X2 *(Mus musculus)* | Lasp1 | XP_006532405.1 | 271 | 53 | 4 | 25.82/5.68 | 1.29 |
| 49 | malate dehydrogenase, cytoplasmic isoform Mdh1 *(Mus musculus)* | Mdh1 | NP_032644.3 | 511 | 28 | 5 | 36.66/6.16 | 1.11 |
| 51 | annexin A1*(Mus musculus)* |  | ACG75704.1 | 1020 | 59 | 8 | 39.01/6.97 | 1.48 |
| 52 | annexin III *(Mus musculus)* | Anxa3 | CAA04887.1 | 859 | 56 | 7 | 36.52/5.33 | 1.62 |
| 53 | annexin A8 isoform 1 *(Mus musculus)* | Anxa8 | NP_038501.2 | 747 | 62 | 8 | 36.93/5.68 | 2.06 |
| 54 | annexin A4 *(Mus musculus)* | Anxa4 | NP_038499.2 | 736 | 57 | 8 | 36.18/5.43 | 1.36 |
| 55 | Chain A, Green Fluorescent Protein From Aequorea Victoria, Mutant | Gfp | pdb\|1EMC\|A | 372 | 55 | 4 | 26.83/5.67 | 6.65 |
| 56 | Chain A, Green Fluorescent Protein Ground States: The Influence Of A Second Protonation Site Near The Chromophore | Gfp | pdb\|2H9W\|A | 473 | 54 | 6 | 26.93/5.46 | 2.38 |
| 57 | PREDICTED: voltage-dependent anion-selective channel protein 1  *(Tupaia chinensis)* | Vdac1 | XP_014439074.1 | 977 | 72 | 7 | 30.87/8.62 | 1.19 |
| 58 | Chain A, Green Fluorescent Protein From Aequorea Victoria, Mutant | Gfp | pdb\|1EMC\|A | 505 | 48 | 4 | 26.83/5.67 | 67.26 |
| 59 | not identified |  |  |  |  |  |  | 1.65 |
| 60 | Prohibitin *(Mus musculus)* | Phb | NP_032857.1 | 491 | 55 | 5 | 29.86/5.57 | 1.90 |
| 61 | phosphoglycerate mutase 1 *(Mus musculus)* | Pgam1 | NP_075907.2 | 201 | 36 | 3 | 28.93/6.67 | 1.15 |
| 62 | enoyl-CoA hydratase, mitochondrial precursor *(Mus musculus)* | Echs1 | NP_444349.1 | 287 | 30 | 3 | 31.85/8.76 | 1.23 |
| 63 | phosphoglycerate mutase 1 *(Mus musculus)* | Pgam1 | NP_075907.2 | 665 | 78 | 5 | 28.93/6.67 | 1.18 |
| 64 | heat shock protein HSP27 *(Mus musculus)* | Hspb1 | AAA18335.1 | 559 | 58 | 5 | 22.94/6.45 | 1.13 |
| 65 | Thioredoxin *(Mus musculus)* | Txn | NP_035790.1 | 394 | 89 | 4 | 12.01/4.80 | 3.55 |
| 66 | galectin-7 *(Mus musculus)* | Lgals7 | NP_032522.2 | 609 | 87 | 6 | 15.25/6.37 | 1.81 |
| 67 | PREDICTED: elongation factor 1-delta isoform X9 *(Mus musculus)* | Eef1d | XP_006521335.1 | 563 | 46 | 5 | 34.95/5.15 | 1.19 |
| 68 | serpin B8 isoform 1 *(Mus musculus)* | Serpinb8 | NP_035589.1 | 350 | 46 | 4 | 42.75/6.05 | 1.25 |
| 69 | heat shock protein HSP 90-alpha *(Mus musculus)* | Hsp90aa1 | NP_034610.1 | 683 | 44 | 7 | 85.13/4.93 | 1.18 |

**Table S2**

Experimental set up for CyDye ^TM^ labeling of four Ad-Foxn1 (1-4) and four Ad-GFP (1-4) samples with the incorporation of a pooled internal standard.

|  | Cy2 | Cy3 | Cy5 |
| --- | --- | --- | --- |
| Gel 1 | 50 µg Pooled Std. | 50 µg sample 1 Ad-Foxn1 | 50 µg sample 1 Ad -GFP |
| Gel 2 | 50 µg Pooled Std. | 50 µg sample 2 Ad-Foxn1 | 50 µg sample 2 Ad-GFP |
| Gel 3 | 50 µg Pooled Std. | 50 µg sample 3 Ad -GFP | 50 µg sample 3 Ad-Foxn1 |
| Gel 4 | 50 µg Pooled Std. | 50 µg sample 4 Ad -GFP | 50 µg sample 4 Ad-Foxn1 |
|  | | | |

**Table S3**

Functional profiling of proteins differentially abundant between keratinocytes transfected with Ad-Foxn1 and Ad-GFP.

| **Molecular function** | **Corrected p-value** | **Proteins** |
| --- | --- | --- |
| Calcium-dependent fhospholipid binding | 2.34∙10^-3^ | Anaxa1, Anaxa3, Anaxa8, Anaxa4 |
| Protein binding | 4.73∙10^-2^ | Mvp, Actn4, Gsn, Cald1, Plcd1, Msn, Pdpk1, Hspa1a, Hsp90aa1, Hspb1, Pls3, Krt5, Krt7, P4hb, Plin3, Pdlim1, Pcbp1, Lasp1, Anaxa1, Anaxa3, Anaxa8, Anaxa4, Vdac1, Phb, Pgam1, Txn, Eef1d |
| *Protein binding involved in cell adhesion* | 1.53∙10^-4^ | Cald1, Hspa1a, Plin3, Pdlim1, Pcbp1, Lasp1, Anaxa1, Eef1d |
| *Cell adhesion molecule binding* | 1.03∙10^-5^ | Cald1, Msn, Hspa1a, P4hb, Plin3, Pdlim1, Pcbp1, Lasp1, Anaxa1, Eef1d |
| Phospholipase A2 inhibitor activity | 4.80∙10^-2^ | Anaxa1, Anaxa3 |
| Actin binding | 1.33∙10^-2^ | Actn4, Gsn, Cald1, Msn, Pls3, Lasp1, Anaxa8 |
| **Cellular component** |  |  |
| Extracellular region | 1.53∙10^-12^ | Mvp, Actn4, Ecsh1, Gsn, Plcd1, Msn, Hspa1a, Hsp90aa1, Hspb1, Krt5, Krt6a, Krt7, P4hb, Phgdh, Pgd, Serpinb2, Ctsd, Psmd13, Arg1, Pcbp1, Lasp1, Mdh1, Anaxa1, Anaxa3, Anaxa8, Anaxa4, Vdac1, Phb, Pgam1, Txn, Lgals7, Serpinb8 |
| Organelle | 1.08∙10^-3^ | Mvp, Actn4, Aco2, Ecsh1, Gsn, Cald1, Plcd1, Msn, Pdpk1, Hspa1a, Hsp90aa1, Hspb1, Pls3, Krt5, Krt6a, Krt6b, Krt7, P4hb, Phgdh, Plin3, Pgd, Ctsd, Psmd13, Arg1, Pcbp1, Lasp1, Mdh1, Anaxa1, Anaxa3, Anaxa8, Anaxa4, Vdac1, Phb, Pgam1, Txn, Eef1d, Serpinb8 |
|  |  |  |
| **Cont., Table S3** | | |
| Intracellular | 3.00∙10^-3^ | Mvp, Actn4, Aco2, Ecsh1, Gsn, Cald1, Plcd1, Msn, Pdpk1, Hspa1a, Hsp90aa1, Hspb1, Pls3, Krt5, Krt6a, Krt6b, Krt7, P4hb, Plin3, Pgd, Serpinb2, Ctsd, Psmd13, Pdlim1, Arg1, Pcbp1, Lasp1, Mdh1, Anaxa1, Anaxa3, Anaxa8, Anaxa4, Vdac1, Phb, Pgam1, Txn, Eef1d, Serpinb8 |
| Cell Junction | 1.53∙10^-4^ | Actn4, Gsn, Cald1, Msn, Pdpk1, Hspa1a, Hspb1, P4hb, Plin3, Pdlim1, Pcbp1, Lasp1, Anaxa1, Eef1d |
| Myelin sheat | 1.74∙10^-7^ | Aco2, Gsn, Msn, Hsp90aa1, Phgdh, Mdh1, Vdac1, Phb, Pgam1 |
| Cytoskeleton | 2.02∙10^-3^ | Mvp, Actn4, Gsn, Cald1, Msn, Hspa1a, Hspb1, Pls3, Krt5, Krt6a, Krt6b, Krt7, Pdlim1, Lasp1, Mdh1 |
| **Biological function** |  |  |
| Response to stress | 3.40∙10^-2^ | Actn4, Gsn, Pdpk1, Hspa1a, Hsp90aa1, Hspb1, Krt6a, P4hb, Serpinb2, Pdlim1, Arg1, Anaxa1, Anaxa3, Anaxa8, Vdac1, Phb, Txn |
| Carboxylic acid metabolic process | 4.48∙10^-2^ | Aco2, Echs1, P4hb, Phgdh, Pgd, Arg1, Mdh1, Anaxa1, Pgam1 |
| Negative regulation of interleukin-8 secretion | 4.80∙10^-2^ | Anaxa1, Anaxa4 |
| Biological adhesion | 4.99∙10^-3^ | Actn4, Gsn, Msn, Pdpk1, Hspa1a, Hsp90aa1, Hspb1, Plin3, Pdlim1, Pcbp1, Lasp1, Anaxa1, Lgals7 |


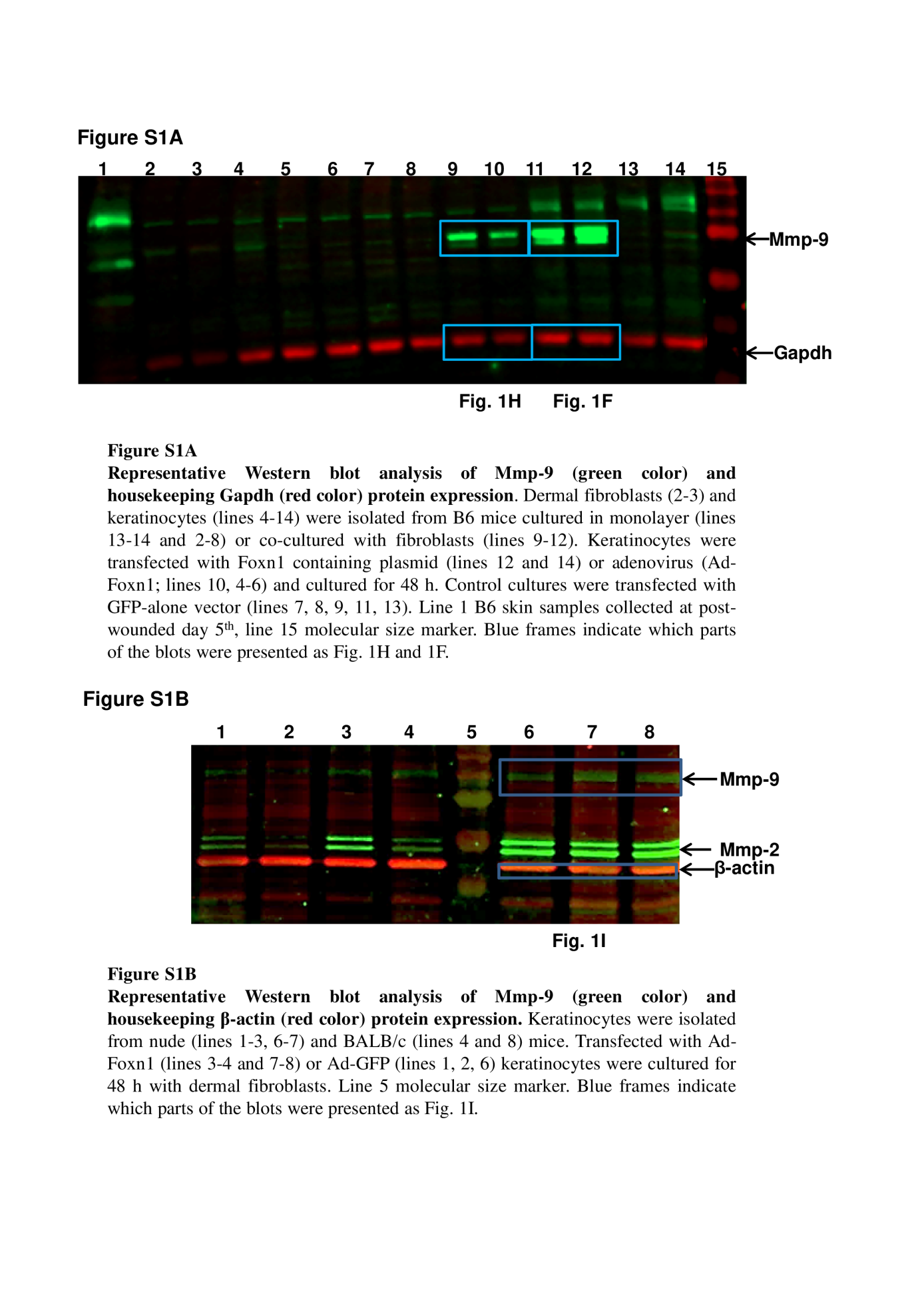


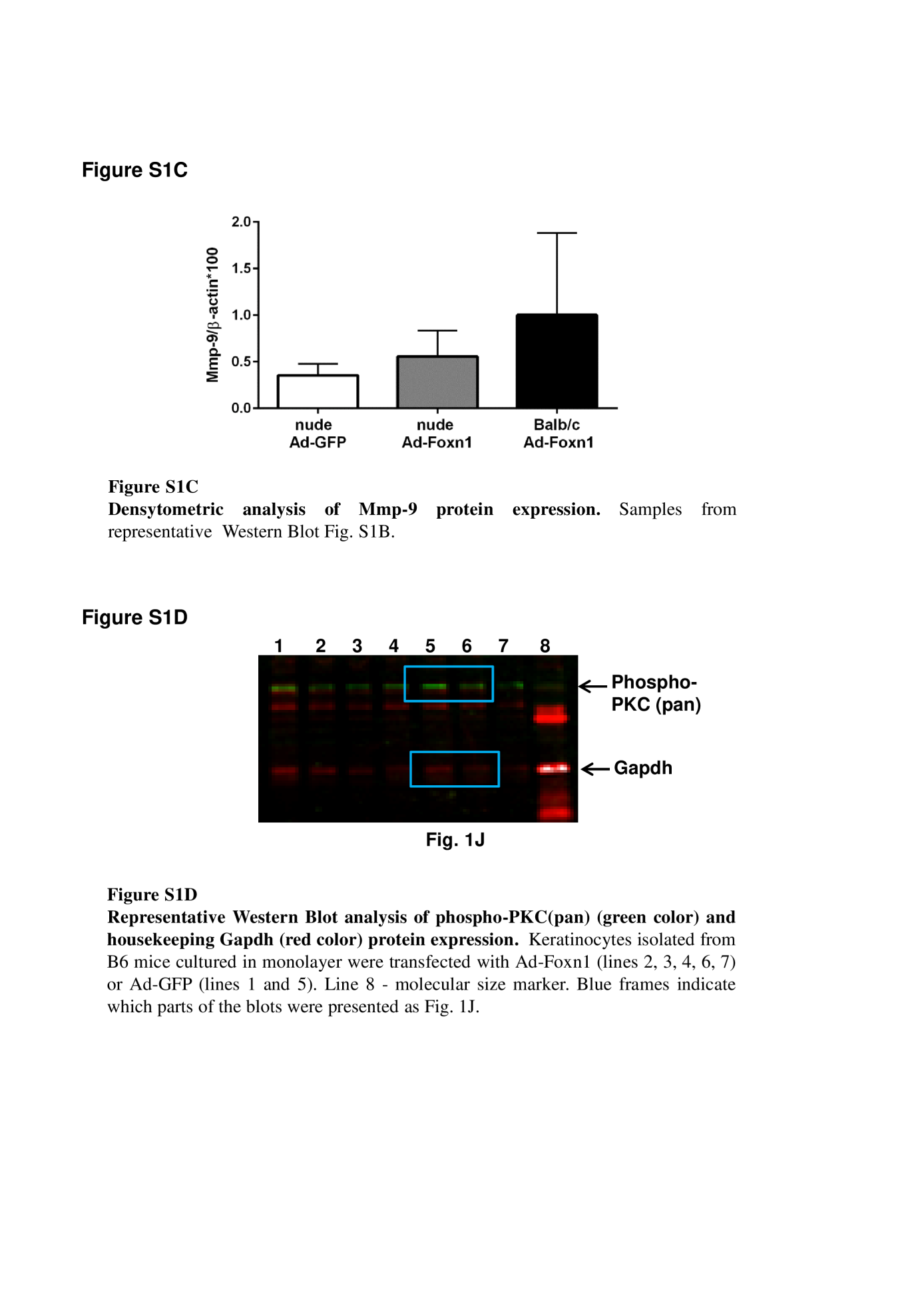


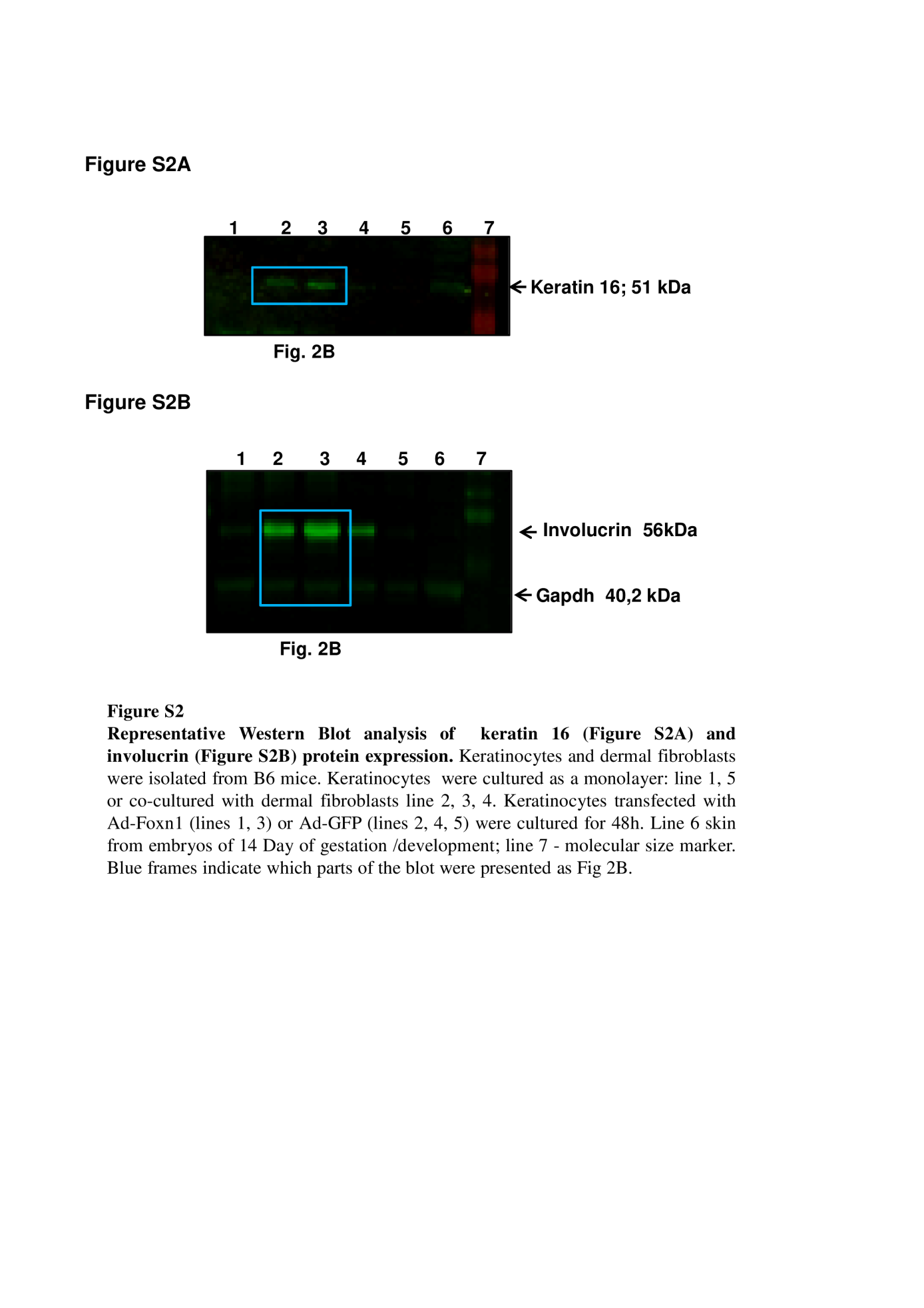


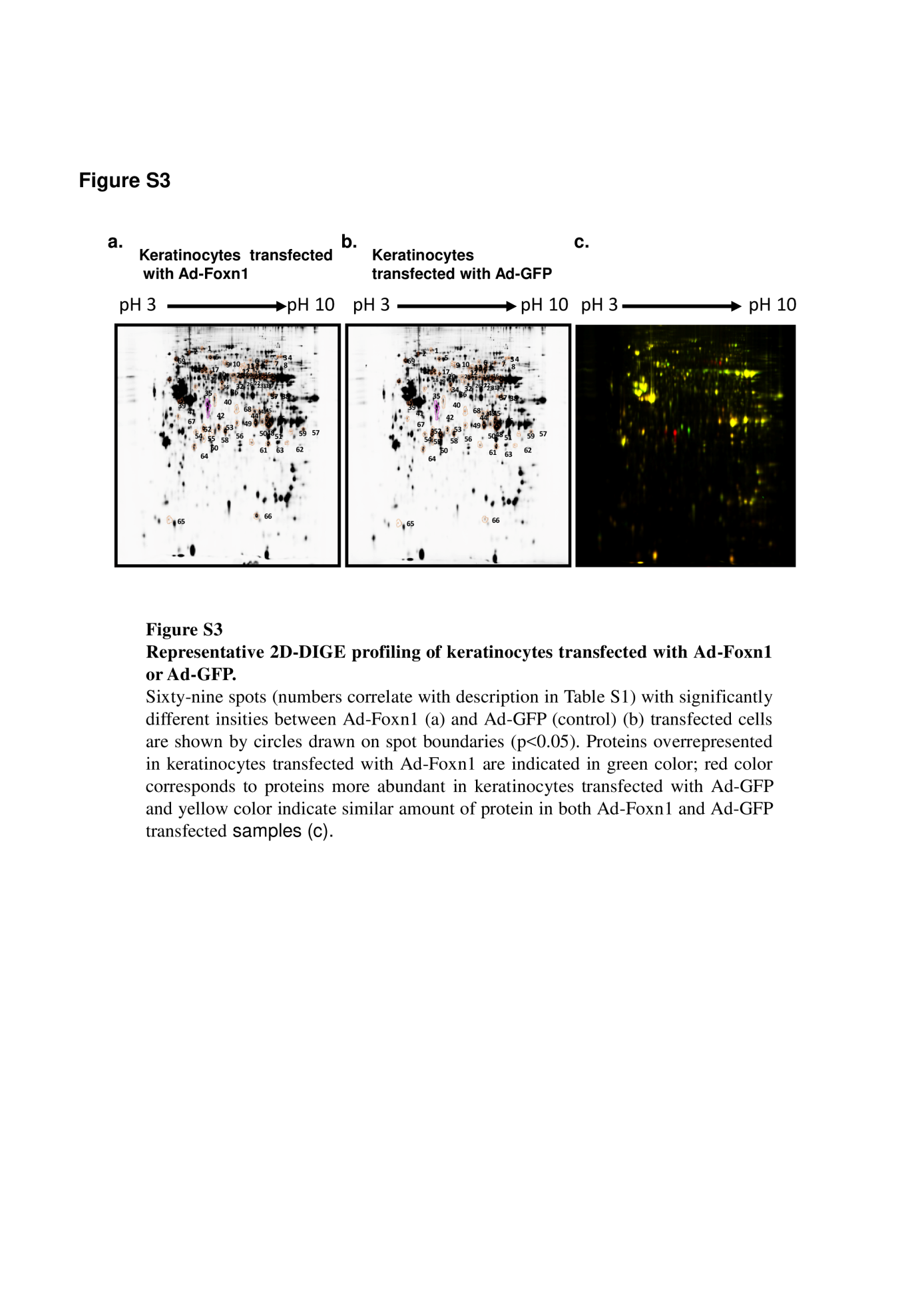


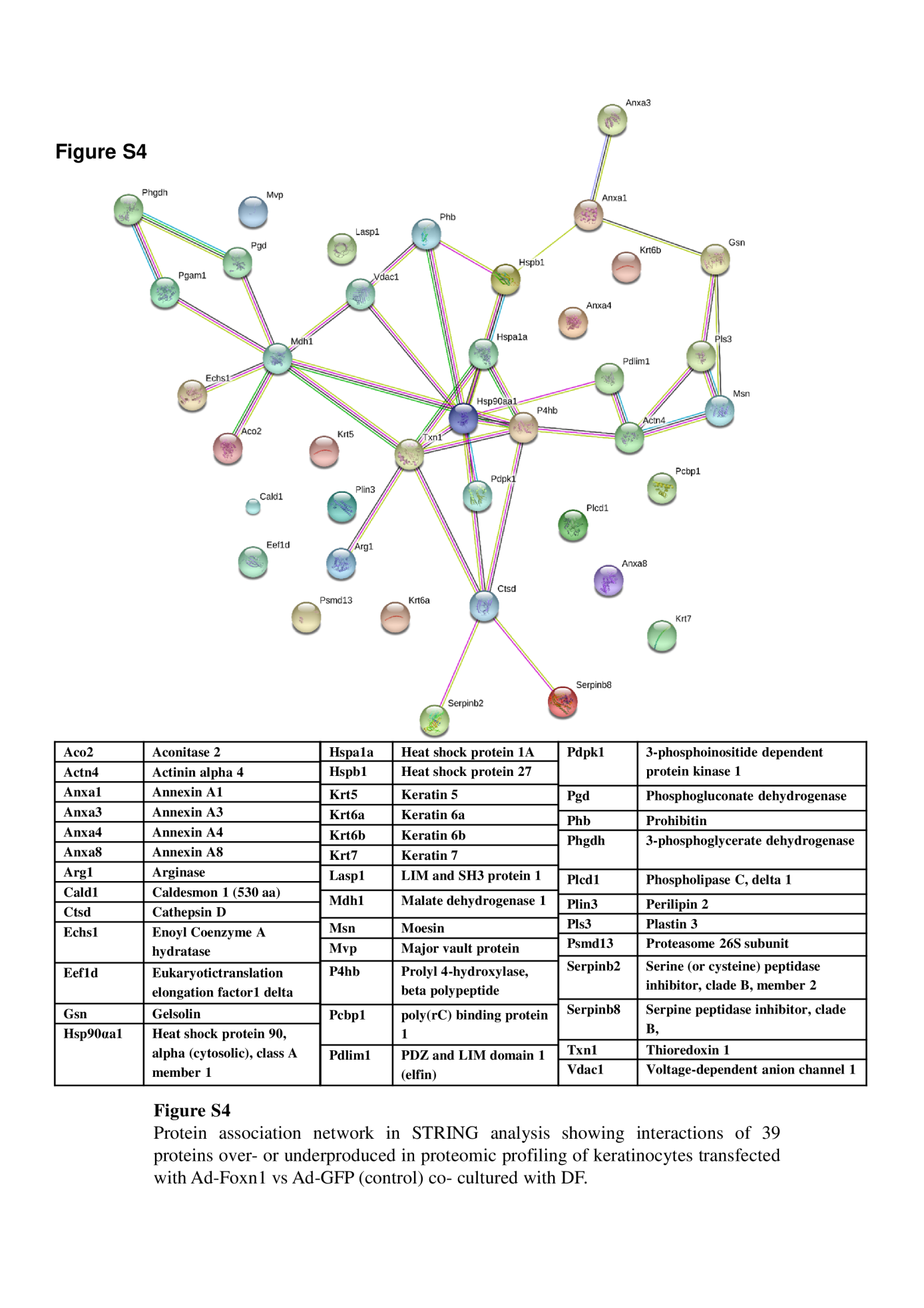


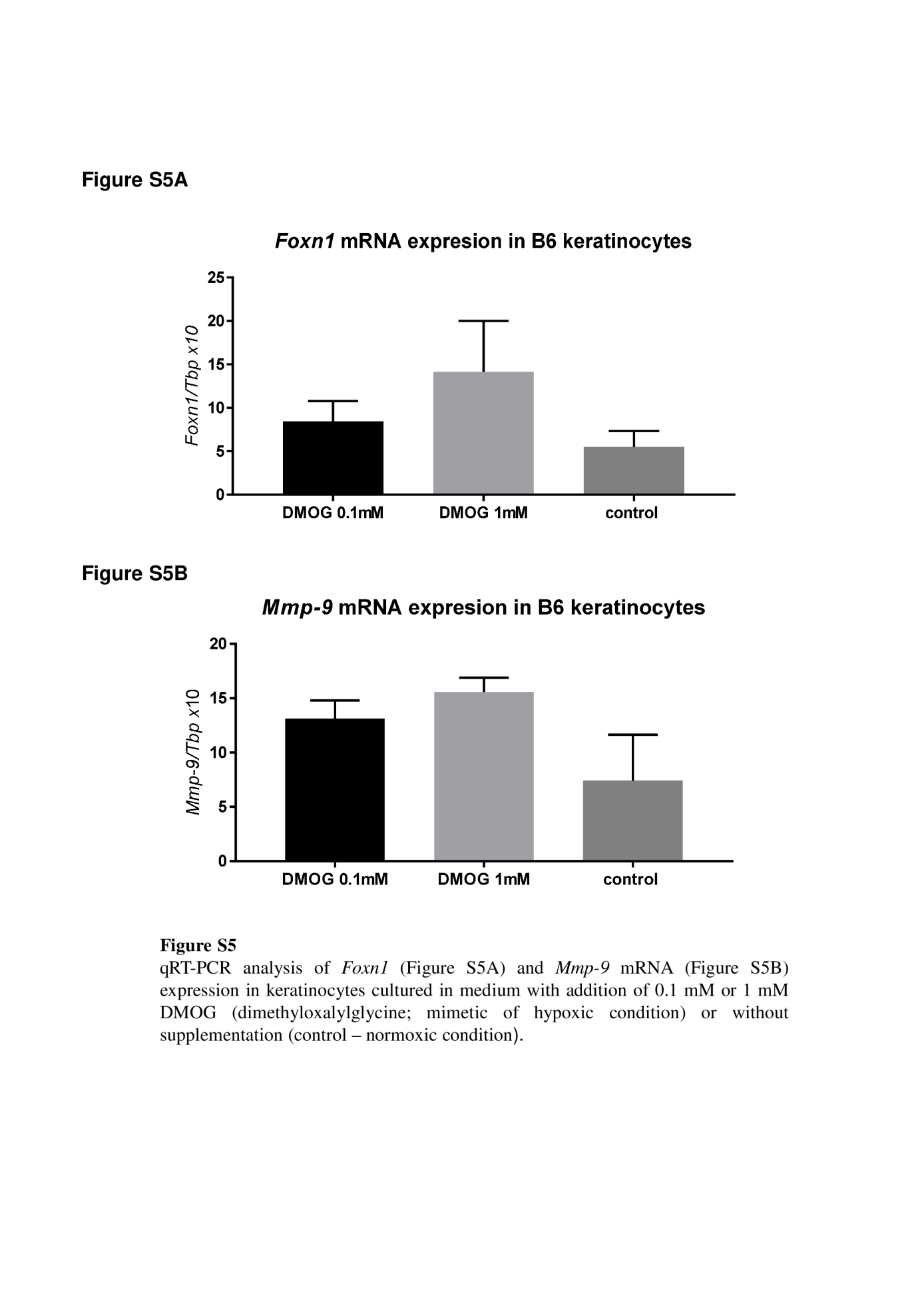


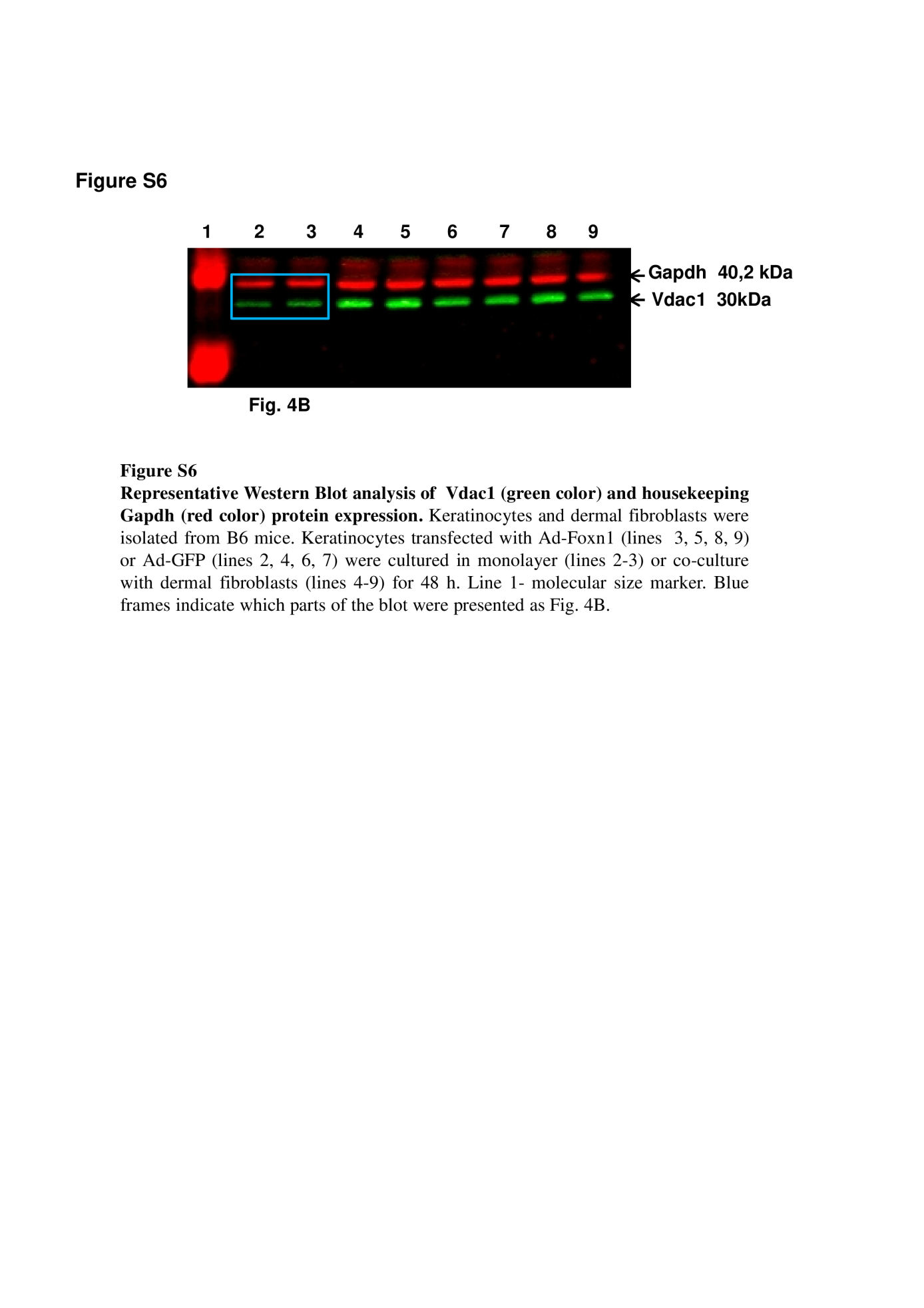


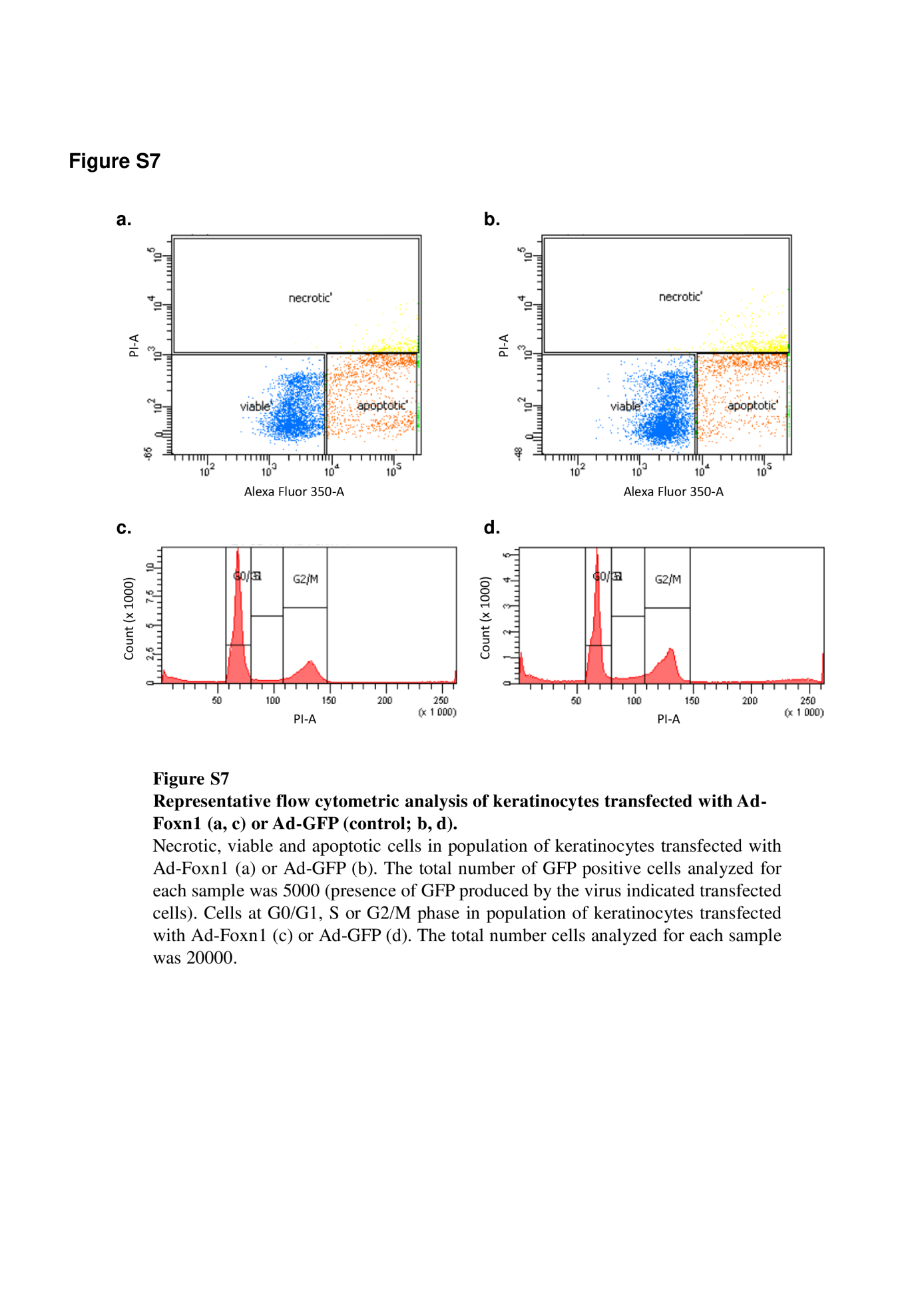

Supplement: Supplementary file 1 — Supplementary Dataset 1 [file 41598_2018_23794_MOESM1_ESM.docx]
